# Combination Treatment with the Vimentin-Targeting Antibody hzVSF and Tenofovir Suppresses Woodchuck Hepatitis Virus Infection in Woodchucks

**DOI:** 10.3390/cells10092321

**Published:** 2021-09-05

**Authors:** Kyle E. Korolowicz, Manasa Suresh, Bin Li, Xu Huang, Changsuek Yon, Bhaskar V. Kallakury, Kyoung-pil Lee, Sungman Park, Yoon-Won Kim, Stephan Menne

**Affiliations:** 1 Department of Microbiology & Immunology, Georgetown University Medical Center, Washington, DC 20057, USA; kek89@georgetown.edu (K.E.K.); ms3687@georgetown.edu (M.S.); Bin.Li@georgetown.edu (B.L.); xh61@georgetown.edu (X.H.); cy33@georgetown.edu (C.Y.); 2 Department of Pathology, Georgetown University Medical Center, Washington, DC 20057, USA; kallakub@georgetown.edu; 3 ImmuneMed, Inc., Chuncheon BioTown, Soyanggang ro 32, Chuncheon-si 24232, Gangwon-do, Korea; philknife@naver.com (K.-p.L.); smpark@immunemed.co.kr (S.P.); ywkim@immunemed.co.kr (Y.-W.K.)

**Keywords:** vimentin, humanized antibody, hzVSF, entry inhibitor, tenofovir alafenamide fumarate, hepatitis B virus, chronic hepatitis B, woodchuck, antiviral efficacy, safety, liver inflammation

## Abstract

Current treatment options for patients infected with hepatitis B virus (HBV) are suboptimal, because the approved drugs rarely induce cure due to the persistence of the viral DNA genome in the nucleus of infected hepatocytes, and are associated with either severe side effects (pegylated interferon-alpha) or require life-long administration (nucleos(t)ide analogs). We report here the evaluation of the safety and therapeutic efficacy of a novel, humanized antibody (hzVSF) in the woodchuck model of HBV infection. hzVSF has been shown to act as a viral entry inhibitor, most likely by suppressing vimentin-mediated endocytosis of virions. Targeting the increased vimentin expression on liver cells by hzVSF after infection with HBV or woodchuck hepatitis virus (WHV) was demonstrated initially. Thereafter, hzVSF safety was assessed in eight woodchucks naïve for WHV infection. Antiviral efficacy of hzVSF was evaluated subsequently in 24 chronic WHV carrier woodchucks by monotreatment with three ascending doses and in combination with tenofovir alafenamide fumarate (TAF). Consistent with the proposed blocking of WHV reinfection, intravenous hzVSF administration for 12 weeks resulted in a modest but transient reduction of viral replication and associated liver inflammation. In combination with oral TAF dosing, the antiviral effect of hzVSF was enhanced and sustained in half of the woodchucks with an antibody response to viral proteins. Thus, hzVSF safely but modestly alters chronic WHV infection in woodchucks; however, as a combination partner to TAF, its antiviral efficacy is markedly increased. The results of this preclinical study support future evaluation of this novel anti-HBV drug in patients.

## 1. Introduction

Infection with hepatitis B virus (HBV) is one of the most serious global public health issues, insofar as approximately 296 million people are chronic carriers of HBV and 820,000 individuals die each year due to HBV-associated liver diseases [1]. Currently approved direct-acting antivirals (i.e., nucleos(t)ide analogues or NAs) targeting the viral polymerase protein rarely achieve sustained loss of HBV surface antigen (HBsAg), with or without subsequent seroconversion to antibodies to HBsAg, which is defined as a “functional cure” [2]. Instead, NAs reduce the number of infected hepatocytes by suppressing viral DNA synthesis and lower the risk of liver disease progression, but require prolonged or even lifelong administration, because the persistent HBV covalently-closed circular (ccc) DNA genome within the nucleus of infected hepatocytes is not targeted, and viral replication typically rebounds after treatment discontinuation [3]. Systemic interferon (i.e., pegylated interferon alpha or PegIFN-α) treatment induces anti-HBV immune responses to control the chronic viral infection, but is sometimes associated with severe side effects, although it mediates a slightly higher rate of functional cure than NAs [2]. Combination treatment with PegIFN-α and NAs increases this rate, albeit it is still limited to less than 10% of patients [2,4]. Thus, novel drugs are wanted for incorporation into already applied treatment regimens for enhancing the HBV cure rate after a finite course of treatment. These new treatment regimens are thought to improve the survival of HBV-infected patients by preventing liver disease progression to cirrhosis and hepatocellular carcinoma (HCC), and subsequent death due to inoperable liver cancer.

HBV is an enveloped virus containing a 3.2 kilobase long, partially double-stranded or relaxed-circular (rc) DNA genome within its nucleocapsid that is formed by the viral core protein. The envelope consists of three glycoproteins, including the large (L), middle (M), and small (S) surface proteins. All proteins have the same C-terminal S domain, but the M and L proteins are extended at the N-terminus to include the additional PreS2 and the PreS1/2 domains, respectively, with the PreS1 domain involved in nucleocapsid binding and cellular entry [5]. HBV entry into hepatocytes initially requires the attachment of virions to heparan sulfate proteoglycans (HSPGs), such as glypican 5, located on the cell surface [6,7,8]. This low affinity binding is mediated by the PreS1 domain and the antigenic loop of the viral L or S proteins, respectively. This interaction then allows a transition of HBV virions to, and subsequent high affinity binding of, the PreS1 domain with the sodium taurocholate co-transporting polypeptide (NTCP) receptor, a hepatocyte-specific bile acid transporter that is integrated in the basolateral membrane of liver cells [9,10]. The predominant expression of NTCP on human liver cells is responsible for the hepatotropism of HBV and further defines the species specificity of the virus. Epidermal growth factor receptor (EGFR) has been identified as a co-receptor for HBV [11]. Thus, HBV virions bound to NTCP and EGFR are internalized into the cytoplasm of hepatocytes by mainly clathrin- and/or EGFR-mediated endocytosis [12,13,14]. HBV virions are subsequently transported from early to late endosomes [15] until they reach the lysosomes/endolysosomes, in which the viral core proteins are cleaved for nucleocapsid dissociation [16]. Movement of early endosomes from the cell periphery to a perinuclear region by microtubules is involved in the maturation process to late endosomes [17].

Therapeutic interference of HBV infection by entry inhibitors has been a focus of investigation in recent years. These inhibitors target the interactions of HBV virions with HSPGs or NTCP by either neutralizing the activities of the viral surface proteins (e.g., antibodies, heparin, suramin, and proanthocyanidin), by binding directly to HSPGs and blocking virion attachment (e.g., synthetic anti-lipopolysaccharide peptides), or by blocking the transport and/or binding functions of the receptor (e.g., cyclosporine A and derivates, ezetimibe, and synthetic lipopeptides) [18,19]. The underlying concept is that continuous blocking of de novo infection of naïve or uninfected, regenerated liver cells and of reinfection of already infected hepatocytes by entry inhibitors during chronic HBV infection results in an overall decline in the fraction of infected cells within the liver. Their prolonged administration then eventually leads to the eradication of persistent HBV infection via natural turnover or immune-mediated clearance of infected hepatocytes in the liver.

Among the HBV entry inhibitors tested so far, Myrcludex B, now bulevirtide (Hepcludex), is the most advanced drug and has entered phase III clinical trials in patients chronically infected with HBV and hepatitis delta virus (HDV). The compound is a synthetic, myristolated peptide derived from the preS1 domain of the HBV L protein and interacts with the NTCP receptor, thereby causing interference with the formation of a functional HBV receptor complex [20]. The peptide has been shown to block and suppress infection of HBV and HDV in vitro in human hepatoma cell lines or in vivo in mouse models, respectively [21]. In addition, combinational treatment with Myrcludex B and systemic PegIFNα-2a demonstrated a clinical benefit in patients with chronic HBV/HDV co-infection in regard to synergistic antiviral effects on HBV-DNA and HDV RNA [22].

Another still experimental HBV entry inhibitor is humanized (hz) virus suppressing factor (VSF), an immunoglobulin G4- (IgG4-) based monoclonal antibody that was derived from the murine homolog by ImmuneMed, Inc. using recombinant techniques [23]. This antibody targets vimentin (VIM) induced by encephalomyocarditis virus and other viruses (called virus-induced VIM or vi-VIM), which is present on the surface of infected but absent on uninfected cells [23]. Viral infections can rearrange intracellular VIM and change its conformation [24,25,26,27] and this may then also allow its presentation at the cell surface by an albeit unknown mechanism. VIM presented at the cell surface is not directly derived from a cell-specific mRNA transcript but is most likely the product of cell-specific posttranslational modification(s) [28]. As a type III intermediate filament, intracellular VIM usually participates in forming the cytoskeleton mainly in cells of mesenchymal origin and provides mechanical stability; however, this protein is also involved in various unrelated physiological processes, including epithelial mesenchymal transition in cancer, inflammation, immune response activation, wound healing, lipogenesis, intracellular signaling, and bacterial and viral infections [29,30,31,32,33]. During viral infections, surface VIM can act as a binding protein; however, intracellular VIM is involved in the internalization of clathrin-coated endosomes, attachment, and transport of capsids along microtubules, and acidification of and capsid release from endosomes [34]. For HBV it has been shown that hzVSF inhibits endocytosis-based entry into human hepatoma cells via the NTCP receptor, possibly through an alteration of intracellular VIM localization [35]. This finding is supported by an unrelated study demonstrating the requirement of VIM for efficient receptor ligand transendocytosis involved in angiogenesis [36]. Furthermore, the activity of hzVSF is not restricted to the HBV/NTCP receptor complex as the antibody also mediates antiviral efficacy and an effect on inflammation induced by the human coronaviruses OC43 and SARS-CoV-2 [37]. Overall, these findings suggest that hzVSF could present a novel option for therapeutic intervention of HBV by preventing viral reinfection of hepatocytes.

We report here the evaluation of hzVSF in the immunocompetent woodchuck animal model of HBV [38]. Infection of neonatal woodchucks with the HBV-like woodchuck hepatitis virus (WHV) resembles the vertical transmission of HBV in humans leading to chronic infection [39]. WHV-mediated immunopathogenesis and liver disease progression in the woodchuck parallels human HBV infection more so than in any other animal model currently available for HBV research [38,40,41,42,43,44,45]. Woodchucks are applied in the assessment of new drugs for the treatment of HBV and HCC, and their preclinical use is predictive of therapeutic efficacy of NAs [46,47] and immunomodulators [48,49] against HBV in patients. However, as WHV uses an unknow receptor other than NTCP for infecting woodchuck hepatocytes [50,51,52], HBV-specific entry inhibitors have not been tested in this animal model so far. In the present study, hzVSF monotreatment in WHV-uninfected and -infected woodchucks was assessed for safety and antiviral efficacy. Since one woodchuck with low WHV replication at treatment initiation experienced a sustained antiviral effect, hzVSF was further evaluated in combination with the NA tenofovir alafenamide fumarate (TAF) for parallel suppression of viral replication during treatment. Thus, the overall hypothesis tested was that treatment of woodchucks with hzVSF, alone and together with TAF, would result in antiviral efficacy against WHV by interfering with the maintenance of chronic infection, and that the antiviral effect induced by combination treatment would be superior over monotreatment.

## 2. Materials and Methods

### 2.1. In Vitro Studies

#### 2.1.1. Human Cell Line and Liver Tissues

Induction of vi-VIM after HBV infection was analyzed in human HepG2 hepatoma cells, while protein presence was assessed in the liver of HBV-uninfected and -infected patients. HepG2 cells were purchased from the Korean Cell Line Bank (Seoul, Republic of Korea) and cultured in Dulbecco Modified Eagle Medium (Biowest, Riverside, MO, USA) until confluence. HBV was collected from the supernatant of HepG2.2.15 cells (Korean Cell Line Bank) and enriched for viral particles with the PEG Virus Precipitation kit (BioVision; Milpitas, CA, USA) by following the manufacturer’s protocol. HepG2 cells were then infected with increasing doses of precipitated HBV (i.e., 50, 150, and 300 μL) and harvested 2-, 4-, 8-, or 12-h post-infection (pi), while noninfected cells served as a control. Cells were subjected to Western blot analysis or immunocytochemistry. A human liver tissue array was purchased from US Biomax (Derwood, MD, USA; cat. no. LV1601) and subjected to immunohistochemistry (IHC) or immunofluorescence staining. The array contains doublets of 80 liver tissues. HBV-uninfected samples include noncancerous liver tissues (35 samples), while HBV-infected samples consist of liver tissues with CHB (13 samples) or cirrhosis (32 samples).

#### 2.1.2. Western Blot

Following HBV infection, HepG2 cells were harvested and resuspended in radioimmunoprecipitation assay (RIPA) lysis buffer (Abcam; Cambridge, MA, USA). Protein concentrations were determined with the Pierce bicinchoninic acid (BCA) Protein Assay kit (Thermo Fisher Scientific, Waltham, MA, USA). Ten µg of proteins were separated by 10% (*w*/*v*) sodium dodecyl sulfate (SDS) polyacrylamide gel electrophoresis and then electrophoretic transferred to polyvinylidene difluoride membranes (Immobilon-P PDVF Membrane; Millipore, Burlington, MA, USA). The membranes were incubated overnight at 4 °C with primary antibodies diluted in Tris-buffered saline (TBS) containing 0.1% (*v*/*v*) Tween-20 and 3% (*w*/*v*) bovine serum albumin. After washing three times with TBS for 10 min, the membranes were incubated with secondary antibodies for 1 h at room temperature. This was followed by washing three times with TBS for 10 min and membrane development with the Pierce Fast Western Blot kit, enhanced chemiluminescence (ECL) Substrate (Thermo Fisher Scientific) by following the manufacture’s protocol. Primary antibodies used were hzVSF for the detection of vi-VIM, a cross-reactive mouse monoclonal antibody to porcine vimentin (V9; Santa Cruz Biotechnology, Dallas, TX, USA; cat. no. sc-6260; 1:1000 dilution) for the detection of intracellular VIM, and a mouse monoclonal antibody to human β-actin (Santa Cruz Biotechnology; cat. no. sc-8432; 1:1000 dilution). Secondary antibodies applied were horseradish peroxidase- (HRP-) conjugated anti-mouse IgG kappa BP (Santa Cruz Biotechnology; cat. no. SC-516102; 1:1000 dilution) and HRP-conjugated anti-human IgG antibody (Sigma-Aldrich, St. Louis, MO, USA; cat. no. AP112P; 1:1000 dilution). Dosimetry was applied to compare VIM signals normalized to the housekeeping protein β-actin. The area of bands was calculated using ImageJ software (National Institutes of Health, Bethesda, MD, USA).

#### 2.1.3. IHC and Immunofluorescence

Human liver tissues were sequentially rehydrated through a graded alcohol series and then deparaffinized with xylene for 2 min. For antigen retrieval, an antigen unmasking solution (Vector Laboratories, Burlingame, CA, USA, cat. no. H-3300) was used by following the manufacturer’s protocol. Tissues were blocked with 0.4% (*v*/*v*) normal goat serum (Vector Laboratories) and 0.4% (*v*/*v*) Triton X-100 in phosphate-buffered saline (PBS) for 1 h at room temperature. Thereafter, tissues were stained with a mouse monoclonal antibody to surface VIM (mouse (m) VSF; 5 µg/mL) and with a rabbit monoclonal antibody to human endogenous VIM (D21H3; Cell Signaling Technology, Danvers, MA, USA; cat. no. 5741; 1:500 dilution) for the detection of vi-VIM or intracellular VIM, respectively. Secondary antibodies applied were Alexa Fluor 488 conjugated goat anti-mouse antibody (Jackson Immuno Research Labs, West Grove, PA, USA; cat. no. 107909; 1:1000 dilution) for mVSF, biotin-conjugated goat anti-mouse antibody (Vector Laboratories; cat. no. BA-1000-1.5; 1:1000 dilution), and Rhodamine Red X conjugated goat anti-rabbit antibody (Jackson Immuno Research Labs; cat. no. 108867; 1:1000 dilution) for D21H3. Unwanted fluorescence signal was removed using Vector TrueVIEW (Vector Laboratories) by following the manufacturer’s protocol. Counterstaining was performed with Hoechst 33,342 (Thermo Fisher Scientific; 1 µg/mL). The biotin-conjugated goat anti-mouse antibody was developed with the VectaStain ABC-HRP kit (Vector Laboratories; 1:1000 dilution) and the DAB Substrate kit (Vector Laboratories) by following the manufacture’s protocol. Counterstaining was performed with Vector Hematoxylin (Vector Laboratories). After dehydration with xylene, tissues were cover-slipped with VectaMount Mounting Medium (Vector Laboratories). Immunohistochemically stained slides were scanned with the Aperio Digital Pathology Slide Scanner AT2 (Leica Biosystems, Buffalo Grove, IL, USA). Immunofluorescence in slides was detected with the Axio Scan.Z1 slide scanner (Carl Zeiss, Oberkochen, Baden-Württemberg, Germany) and the LSM710 confocal microscope (Carl Zeiss).

#### 2.1.4. Immunocytochemistry

HepG2 cells at a concentration of 5 × 10^4^/mL were incubated in a collagen-coated Cell Culture Slide II chamber system (SPL Life Sciences, Pocheon-si, Gyeonggi-do, Republic of Korea). After cell seeding for 24 h, 2% (*v*/*v*) of dimethyl sulfoxide (DMSO) was added to the culture medium for 24 h for enhancing infection with HBV derived from the supernatant of HepG2.2.15 cells and precipitated as described above. After HBV infection for 4 h, cells were fixed with 4% (*v*/*v*) paraformaldehyde in PBS (pH 7.4) containing 1% (*v*/*v*) of glutaraldehyde. Attached cells were stained with mVSF or D21H3 as described above. Secondary antibodies applied were Alexa Fluor 488 conjugated goat anti-mouse antibody for mVSF and Rhodamine Red X conjugated goat anti-rabbit antibody for D21H3 as described above. Counterstaining was performed with Hoechst 33,342 (Thermo Fisher Scientific). Cells were cover-slipped with VECTASHIELD Antifade Mounting Media (Vector Laboratories) and imaged using the Axio Imager A1 microscope (Carl Zeiss).

### 2.2. In Vivo Studies

#### 2.2.1. Investigational Drugs

hzVSF was provided as a frozen solution. A volume sufficient to treat all experimental woodchucks for one week was thawed and diluted in isotonic PBS (Alanza, Aurora, ON, Canada) for obtaining antibody concentrations of 0.1, 4.0, or 16.0 mg/mL. A volume of 1.0 mL hzVSF per kg body weight of an individual, pre-weighed woodchuck was then administered by slow bolus injection via the femoral vein of the right or left hind legs of an anesthetized animal. The intermediate hzVSF dose of 4.0 mg/kg is in the range of the highest antibody dose (i.e., 200 mg) administered to patients with severe COVID-19 symptoms [37]. hzVSF vehicle was isotonic PBS (Alanza) and a volume of 1.0 mL per kg body weight was intravenously (iv) administered to animals as described for hzVSF. TAF was purchased from Selleck Chemicals LLC (Houston, TX, USA). An amount of TAF powder sufficient to treat all experimental woodchucks per day was dissolved in isotonic PBS (G-Biosciences, St. Louis, MO, USA) with 0.5% (*v*/*v*) DMSO for obtaining a concentration of 5.0 mg/mL. Within 30 min after preparation, a volume of 1.0 mL per kg body weight was mixed with an equal amount of woodchuck liquid diet (Dyets, Bethlehem, PA, USA) and orally (po) administered to unanesthetized animals with a dose syringe. The TAF dose was selected based on the 120 mg dose that was safely administered to HBV-infected patients during short-term treatment [53]. For woodchucks, the obtained per-kilogram dose was scaled three-fold to account for differences in metabolic body size [54]. TAF vehicle was isotonic PBS (G-Biosciences) and a volume of 1.0 mL per kg body weight was mixed with woodchuck liquid diet (Dyets) and po administered to animals as described for TAF.

#### 2.2.2. Animals

The eight WHV-uninfected woodchucks and the 24 WHV-infected animals utilized were born and maintained at the animal facilities of Roswell Park Comprehensive Cancer Center (Buffalo, NY, USA). Woodchucks were inoculated at 3 days of age with strain 7 of WHV (WHV7) [39] and raised to adulthood, while regularly tested for verifying the establishment of chronic WHV infection. Woodchucks were transferred to the animal facilities of Georgetown University (Washington, DC, USA) at an age of one (chronic WHV carrier woodchucks) or two years (WHV-uninfected animals). Upon arrival, uninfected animals were confirmed negative for serum WHV DNA, WHV surface antigen (WHsAg), WHV e antigen (WHeAg), and antibodies to WHsAg and WHeAg (anti-WHs and anti-WHe antibodies). Infected animals were confirmed positive for serum WHV DNA, WHsAg, and WHeAg, and negative for anti-WHs and anti-WHe antibodies. Most chronic WHV carrier woodchucks presented with low serum gamma-glutamyl transferase (GGT) activity, an oncogenic marker of HCC in woodchucks [55], and the absence of liver tumors was confirmed by ultrasonography on anesthetized animals. Three woodchucks (i.e., F1854, F1855, and M1856) had elevated serum GGT activity and a smaller liver tumor was detected during the initial ultrasound examination. WHV-uninfected woodchucks were allocated to two groups (Table S1) and randomized within blocks and factors. The block for stratification was sex. The factor was the categorial variable body weight (low, medium, and high). If needed, animals were moved between both groups based on serum liver enzyme levels (GGT, sorbitol dehydrogenase [SDH], aspartate aminotransferase [AST] and alanine aminotransferase [ALT]), for achieving comparable ranges. Chronic WHV carrier woodchucks were allocated to six groups (Table S1) and also randomized within blocks (i.e., sex) and factors (i.e., body weight). If needed, animals were moved between the groups based on other parameters, including pretreatment serum viral markers (WHV DNA, WHsAg, and WHeAg) and liver enzyme levels, for achieving comparable ranges within each group. Animal procedures involving hzVSF vehicle and hzVSF iv administration, blood collection, percutaneous liver biopsy, and euthanasia were performed under isoflurane inhalation and/or ketamine/xylazine im injection anesthesia. Animal research staff was not blinded in regard to treatment administration and animal procedures. However, laboratory research staff was blinded to animal group/treatment allocation during sample processing and analysis.

#### 2.2.3. Study Design

**Figure 1:**
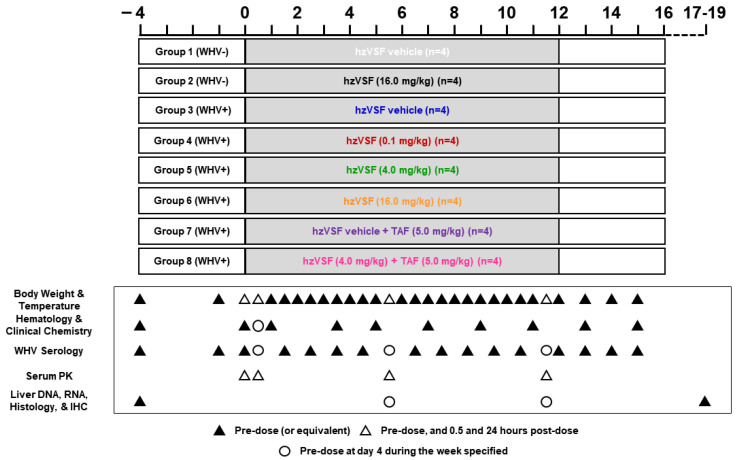
Design of the three hzVSF treatment studies in woodchucks. In the first study, WHV-uninfected woodchucks received iv hzVSF vehicle (Group 1) or hzVSF at a dose of 16.0 mg/kg (Group 2) twice-weekly for 12 weeks. In the second study, chronic WHV carrier woodchucks were administered iv hzVSF vehicle (Group 3) or hzVSF at doses of 0.1 mg/kg (Group 4), 4.0 mg/kg (Group 5), or 16.0 mg/kg (Group 6), respectively, twice-weekly for 12 weeks. In the third study, chronic WHV carrier woodchucks received iv hzVSF vehicle (Group 7) or hzVSF at a dose of 4.0 mg/kg (Group 8) twice-weekly for 12 weeks, together with po TAF at a dose of 5.0 mg/kg once-daily for 12 weeks. After treatment cessation, animals were followed for additional 4 weeks until the EOS in week 16. The terminal collection of liver and other organs was performed at euthanasia during weeks 17–19. Arrows indicate the time of measurements for the specific parameters listed. Abbreviation: IHC, immunohistochemistry.

A total of three studies was performed (Figure 1): The first study included eight WHV-uninfected woodchucks and two groups. Group 1 animals (n = 4) were iv treated with hzVSF vehicle twice-weekly for 12 weeks, while Group 2 animals (n = 4) received iv hzVSF twice-weekly for 12 weeks at a dose of 16 mg/kg. The second study involved 16 chronic WHV carrier woodchucks and four groups. Group 3 animals (n = 4) were iv treated with hzVSF vehicle twice-weekly for 12 weeks. Group 4, Group 5, and Group 6 animals (each n = 4) received iv hzVSF twice-weekly for 12 weeks at doses of 0.1, 4.0, or 16.0 mg/kg, respectively. The third study involved additional eight chronic WHV carrier woodchucks and two groups. Group 7 animals (n = 4) were iv treated with hzVSF vehicle twice-weekly for 12 weeks in combination with daily po TAF for 12 weeks at a dose of 5.0 mg/kg. Group 8 animals (n = 4) received iv hzVSF twice-weekly for 12 weeks at a dose of 4.0 mg/kg in combination with daily po TAF for 12 weeks at a dose of 5.0 mg/kg. After treatment cessation, all woodchucks were followed for additional four weeks until the end of the study (EOS) at week 16 and then euthanized during weeks 17–19.

Changes in serum and liver viremia and antigenemia levels, as well as the elicitation of anti-WHs and anti-WHe antibodies, were evaluated for determining antiviral effects of the mono and combination treatment regimens. Changes in sinusoidal and lobular hepatitis, intrahepatic cluster of differentiation (CD) 3+ T-cells and macrophages, and regenerating hepatocytes positive for the proliferation marker Ki67 were assessed for determining liver inflammation and replenishment. Presence of vi-VIM in the liver of selected WHV-uninfected and chronic WHV carrier woodchucks was assayed before treatment initiation. Clinical observations and changes in body weight, body temperature, and hematology and clinical chemistry parameters, as well as necropsy observations, organ weights, and organ histopathology, were evaluated for determining drug safety. Mortality associated with hzVSF and/or TAF treatment was not observed. One woodchuck died and two animals underwent scheduled euthanasia during the treatment period. Woodchuck M1814 (Group 4) experienced an adverse, uncontrollable reaction against the ketamine/xylazine anesthetic administered during the liver biopsy procedure at week 12 and died shortly thereafter. Woodchucks F1854 (Group 4) and F1855 (Group 5) were euthanized during week 10 due to the development of large liver tumors/end-stage HCC.

#### 2.2.4. Blood Collection

Blood samples for testing serology, hematology, and clinical chemistry were obtained via femoral venipuncture from anesthetized woodchucks prior to iv administration of hzVSF vehicle or hzVSF and po dosing with TAF (Figure 1). Blood samples for serology were collected during pretreatment at week −4 and T0, and then weekly during the treatment and follow-up periods. Blood samples for hematology and clinical chemistry were obtained during pretreatment at week −4 and T0, then during treatment at day 4 and at weeks 2, 4, 6, 8, 10, and 12, and thereafter during follow-up at weeks 14 and 16. Blood samples for assessing hzVSF pharmacokinetics were collected during pretreatment at T0, and then during treatment at day 3 and at weeks 6 and 12. Blood collection here was performed before the administration of hzVSF vehicle or hzVSF and TAF, then at 0.5 h post-dose, and thereafter the next day at 24 h post-dose.

#### 2.2.5. Liver Tissue Collection

Ultrasound-guided, percutaneous liver biopsies for assessing WHV nucleic acids, WHV antigens, cell subsets, and histology were obtained from anesthetized woodchucks prior to administration of hzVSF vehicle or hzVSF and TAF (Figure 1). Liver tissues were collected during pretreatment at week −4, then during treatment at weeks 6 and 12, and after the EOS at euthanasia during weeks 17–19. Liver samples for analyzing WHV DNA replicative intermediate (RI), WHV cccDNA, and WHV RNA levels were immediately placed into liquid nitrogen and stored at −80 °C. Liver samples for determining cytoplasmic WHcAg, membranous WHsAg, CD3+ T-cells, macrophages, Ki67+ hepatocytes, vi-VIM, and disease progression were stored in 10% (*v*/*v*) phosphate-buffered formalin and subsequently embedded into paraffin.

#### 2.2.6. Serum WHV Parameters

Serum WHV DNA levels were assayed quantitatively by slot-blot hybridization and real-time PCR, as described previously [54,56]. The lower limit of detection (LLOD) of the PCR assay was 600 WHV genomic equivalents (ge) or copy numbers per mL serum. Serum WHsAg levels were assayed quantitatively by an enzyme-linked immunosorbent assay (ELISA) comparable to the assay described previously [56,57]. The LLOD of the ELISA was 5 ng WHsAg per mL serum. Serum WHeAg levels were assayed qualitatively using a cross-reactive ELISA (DiaSorin, Minneapolis, MN, USA) by following the manufacturer’s protocol. Results were obtained as an optical density read out, and a value of ≤0.055–0.057 optical density units (ODU) indicated absence of WHeAg in this study. Serum anti-WHs antibody titers were assayed quantitatively using an established enzyme immunoassay (EIA), as described previously [56,57]. The LLOD of the EIA using a 1:100 sample dilution was 100 standard units (StdU) per mL serum. Serum anti-WHe antibody levels were assayed qualitatively using a cross-reactive ELISA (DiaSorin) by following the manufacturer’s protocol. An ODU value of ≥2.19 or ≥2.52 (i.e., sample ODU value at pretreatment (T0) minus sample ODU value in a given study week) indicated presence of anti-WHe antibodies in this study.

#### 2.2.7. Liver WHV Parameters

Intrahepatic WHV DNA RI and WHV cccDNA levels were assayed quantitatively by Southern blot hybridization, while intrahepatic WHV RNA levels were determined quantitatively by Northern blot hybridization, as described previously [54,56]. Woodchuck β-actin was used for normalization of WHV nucleic acid concentrations. Both hybridization assays provided results spanning up to >1 and >2 orders of magnitude of detection for WHV RNA and WHV cccDNA or WHV DNA RI species, respectively. The LLOD for both assays was 2 pg WHV DNA or WHV RNA per µg cellular nucleic acids.

#### 2.2.8. Hematology and Clinical Chemistry Parameters

Blood samples for hematology and serum clinical chemistry were analyzed at the Animal Health Diagnostic Center of Cornell University (Ithaca, NY, USA) using parameters validated for woodchucks [55,58]. Hematology parameters included white blood cells, segmented neutrophils, banded neutrophils, lymphocytes, monocytes, eosinophils, basophils, red blood cells, hemoglobin, hematocrit, mean cell volume, mean cell hemoglobin, mean cell hemoglobin concentration, red cell distribution width, platelet count, and mean platelet volume. Clinical chemistry parameters included alkaline phosphatase (ALP), ALT, AST, GGT, SDH, sodium, potassium, chloride, bicarbonate, anion gap, sodium/potassium ratio, urea, creatinine, calcium, phosphate, magnesium, total protein, albumin, globulin, albumin/globulin ratio, glucose, total bilirubin, direct bilirubin, indirect bilirubin, amylase, cholesterol, creatine kinase, iron, total iron binding capacity, percent saturation, lipemia, hemolysis, and icterus.

#### 2.2.9. IHC

Paraffin-embedded liver tissues were sectioned (5 microns), deparaffinized with xylene, and rehydrated through a graded alcohol series at the Histopathology & Tissue Shared Resource (HTSR) Laboratory of Georgetown University. Heat induced epitope retrieval was performed by immersing tissue sections in buffer (10 mM citrate with 0.05% (*v*/*v*) Tween, pH 6.0) for 20 min at 98 °C. Immunohistochemical staining was accomplished with the VectaStain kit (Vector Laboratories) by following the manufacturer’s protocol. Sections were then treated with 3% (*v*/*v*) hydrogen peroxide and endogenous biotin was blocked with an avidin/biotin blocking kit (Thermo Fisher Scientific). Sections were treated subsequently with 10% (*v*/*v*) goat serum and stained with polyclonal rabbit antibodies to WHcAg (1:400 dilution) or WHsAg (1:350). Other sections were stained with a cross-reactive mouse monoclonal antibody to human CD3 (Novocastra/Leica Biosystems, Richmond, IL, USA; cat. no. CD3-565-L-CE; 1:200 dilution) or a cross-reactive mouse monoclonal antibody to human Ki67 (Dianova, Hamburg, Germany; cat. no. DIA-670-P05; 1:135 dilution). Theses sections were stained thereafter with biotin-conjugated anti-rabbit or anti-mouse secondary antibodies (Vector Laboratories) using VectaStain ABC reagent (Vector Laboratories), and 3,3′-diaminobenzidine (DAB) chromogen (Dako, Santa Clara, CA, USA).

For other tissue sections, heat induced epitope retrieval was performed with the Target Retrieval Solution, Low pH (Dako) in the PT Link instrument (Dako). Sections were treated with 3% (*v*/*v*) hydrogen peroxide and subsequently with 10% (*v*/*v*) goat serum and then stained for macrophages with a cross-reactive rat monoclonal antibody to mouse MAC2 (LifeSpan BioSciences, Seattle, WA, USA; cat. no. LS-C62936; 1:200 dilution). These sections were stained thereafter with an HRP-labeled polymer (Vector Laboratories; cat. no. MP-7444-15) and DAB chromogen.

Additional tissue sections from selected animals were stained with hzVSF (40 mg/mL, 1:6000 dilution) followed by an HRP-conjugated monoclonal antibody to human IgG4 (Bethyl Laboratories, Montgomery, TX, USA; cat. no. A80-115P; 1:200 dilution).

In all cases, tissue sections were counterstained with hematoxylin (1:17 dilution), blued in 1% (*w*/*v*) ammonium hydroxide, dehydrated, and mounted with acrymount medium (Thermo Fisher Scientific). All tissue sections were subsequently examined by a board-certified pathologist (BVK) under a light microscope and the percentage of stained hepatocytes/intrahepatic cell subsets determined.

#### 2.2.10. Histology

Paraffin-embedded liver and organ tissues were sectioned (5 microns) and stained with hematoxylin and eosin at the HTSR Laboratory. Tissue sections were then examined by BVK under a light microscope. Liver disease progression, including portal and sinusoidal hepatitis, bile duct proliferation, steatosis, fibrosis, and necrosis, was assessed using criteria developed for woodchuck liver [59,60], as well as by applying the METAVIR scale for scoring human liver. Additional organs obtained at necropsy and subsequently examined by BVK for histopathological changes related to treatment with hzVSF and/or TAF included mesenteric lymph nodes, inguinal skin, inguinal mammary glands, thymus, heart, lungs, lung bronchi, lung trachea, spleen, kidneys, pancreas, stomach, duodenum, jejunum, ileum, cecum, colon, rectum, adrenal glands, urinary bladder, uterus, ovaries, vagina, prostate, testes, epididymis, seminal vesicle, thyroid, parathyroid, submandibular lymph nodes, salivary glands (submandibular, sublingual, and parotid glands), esophagus, brain, pituitary, eye, optic nerve, harderian gland, tongue, bone marrow (sternum and femur), and thoracic spinal cord. Furthermore, selected organs were weighed at necropsy for detecting atrophic or hypertrophic changes related to treatment with hzVSF and/or TAF, including brain, pituitary, heart, lungs, liver, gallbladder, spleen, kidneys, adrenal glands, thymus, prostate, testes, uterus, and ovaries.

#### 2.2.11. Statistical Analysis

All data was inspected for consistency and completeness before statistical analysis. Values below an individual assay LLOD were replaced by the corresponding detection limit (i.e., 600 ge/mL for WHV DNA, 5 ng/mL for WHsAg, 100 StdU/mL for anti-WHs antibody, and 2 pg WHV DNA or WHV RNA/µg cellular nucleic acids). Data for serum WHV DNA and WHsAg were transformed to a log_10_ scale and arithmetically averaged prior to statistical analysis. Intra- and inter-group statistical comparisons were performed using unpaired Student’s *t*-test with equal variance at each timepoint of the study for changes in the following mean parameters: body weight, body temperature, hematology, clinical chemistry, serum and liver WHV markers, liver IHC, liver pathology, and organ weights. *p* values < 0.05 were considered statistically significant.

## 3. Results

### 3.1. Vimentin Was Induced by HBV In Vitro and vi-VIM Presence Was Increased in the Liver of HBV-Infected Patients and WHV-Infected Woodchucks

**Figure 2:**
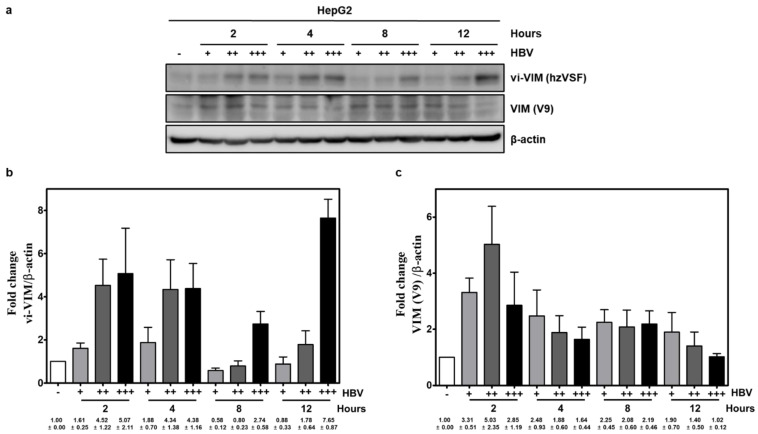
vi-VIM is induced by HBV in human hepatoma cells. (**a**) HepG2 cells were infected with increasing doses of precipitated HBV derived from the supernatant of HepG2.2.15 cells (i.e., + = 50 μL, ++ = 150 μL, and +++ = 300 μL of the HBV precipitate). Changes in vi-VIM level were detected with the humanized hzVSF antibody at 2, 4, 8, and 12 h pi. Parallel changes in intracellular VIM levels were assayed with the V9 antibody. Changes in protein signal were normalized to β-actin and averaged for three replicates, and are presented (**b**) for vi-VIM and (**c**) intracellular VIM as a mean ± standard error of the mean.

For testing VIM upregulation by HBV, HepG2 cells were infected with increasing doses of HBV and vi-VIM was detected via binding to the humanized hzVSF antibody by Western blot (Figure 2). hzVSF-bound vi-VIM increased dose-dependently during 2–12 h pi. Intracellular VIM, as detected by the V9 antibody, also increased initially with all three HBV doses, but its endogenous presence declined over time, and especially at 4 and 12 h pi with the highest HBV dose.

**Figure 3:**
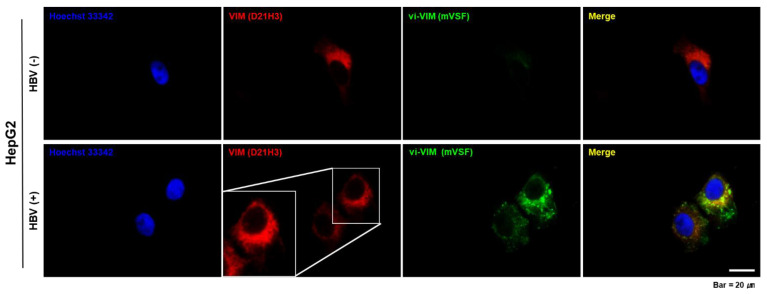
vi-VIM is strongly induced by HBV after infection of human hepatoma cells. HepG2 cells were infected with 50 µL of precipitated HBV derived from the supernatant of HepG2.2.15 cells. Changes in intracellular VIM (red color) and vi-VIM (green color) were detected 4 h pi by immunocytochemistry staining with D21H3 or mVSF antibodies, respectively. Merging of both stains (yellow color) indicated a perinuclear colocalization of intracellular VIM and vi-VIM. Staining with Hoechst 33,342 was used to detect cell nuclei (blue color).

Immunocytochemistry staining was further applied to HBV-uninfected and -infected HepG2 cells (Figure 3). Intracellular VIM, as detected by the D21H3 antibody, increased 4 h pi with 50 µL of the HBV precipitate and was localized around the cell nuclei. mVSF antibody-bound vi-VIM was strongly detected after HBV infection and colocalized with intracellular VIM in the same perinuclear region. Compared to intracellular VIM, vi-VIM appeared concentrated in several areas and also present in form of filamentous structures.

**Figure 4:**
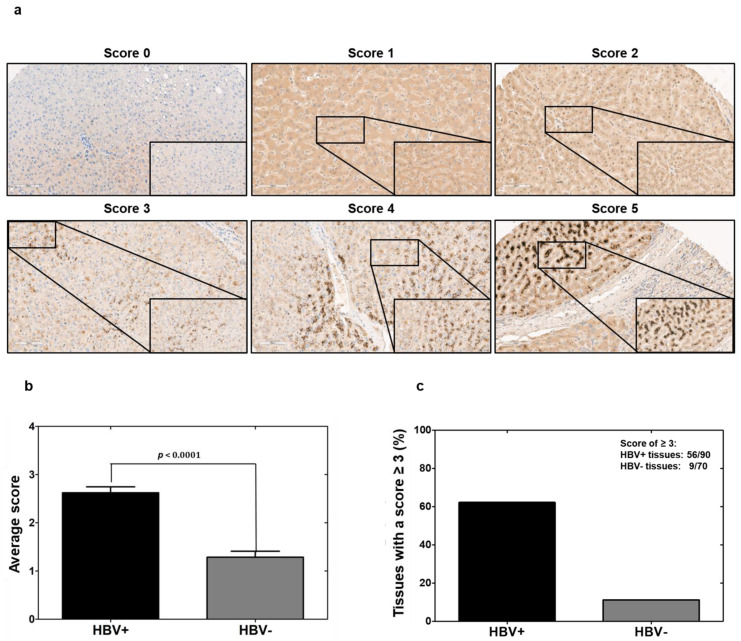
The presence of vi-VIM is significantly increased in HBV-infected liver. (**a**) A human liver tissue array was stained with mVSF by IHC and the staining intensity and distribution of antibody-bound vi-VIM were scored on a 0–5 scale. (**b**) Comparison of the average scores between HBV-uninfected and -infected liver tissues. (**c**) Comparison of percentages of HBV-uninfected and -infected liver tissues with a score of ≥3.

For confirming the in vitro results on HBV-induced vi-VIM upregulation, HBV-uninfected and -infected human liver tissues with progressing disease (i.e., CHB, and cirrhosis) were stained with the mVSF antibody during IHC or immunofluorescence. Staining intensity and distribution of mVSF-bound vi-VIM after IHC was scored on a 0–5 scale (Figure 4). The comparison of average scores revealed that the vi-VIM presence in HBV-infected liver was significantly increased over HBV-uninfected liver (i.e., the score nearly doubled from 1.4 to 2.7). In addition, 65.6% (56/90) of HBV-infected liver tissues were assigned with a score of ≥3, compared to the 12.9% (9/70) of HBV-uninfected liver tissues.

**Figure 5:**
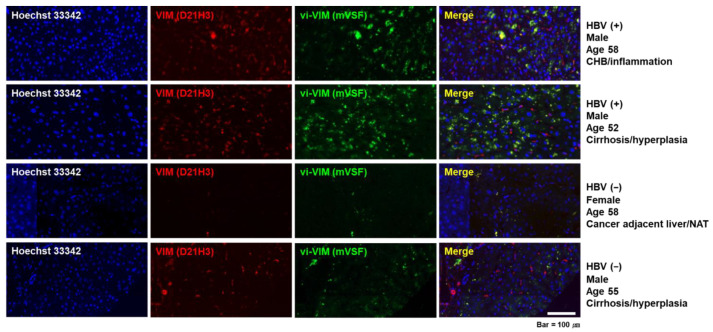
The presence of vi-VIM is strongly increased in HBV-infected liver. A human liver tissue array was used for detecting changes in the presence of intracellular VIM (red color) and vi-VIM (green color) by immunofluorescence staining with D21H3 or mVSF antibodies, respectively. Merging of both stains (yellow color) indicated a perinuclear colocalization of intracellular VIM and vi-VIM in many hepatocytes. Hoechst 33,342 staining was used to detect cell nuclei (blue color). Representative pictures of two HBV-infected and two HBV-uninfected livers are shown. Abbreviation: NAT, nonalcoholic liver tumor.

The above human liver tissues were also subjected to immunofluorescence staining with the mVSF and D21H3 antibodies. Compared to HBV-uninfected liver tissues, the presence of intracellular VIM and vi-VIM was strongly increased in HBV-infected liver and both proteins colocalized around the nucleus in many hepatocytes (Figure 5). In addition, the presence of both proteins was increased during progressing liver disease in both HBV settings (i.e., more in cirrhotic HBV-infected liver than in CHB and somewhat more in cirrhotic HBV-uninfected liver than in normal liver adjacent to a nonalcoholic liver tumor). Staining intensity and distribution of antibody-bound vi-VIM after immunofluorescence of all liver tissues was again scored on a 0–5 scale and the average scores compared (data not shown). The presence of vi-VIM in HBV-infected liver was significantly increased over HBV-uninfected liver (i.e., with a score of 2.62 ± 0.12 versus a score of 1.29 ± 0.13; *p* < 0.0001).

**Figure 6:**
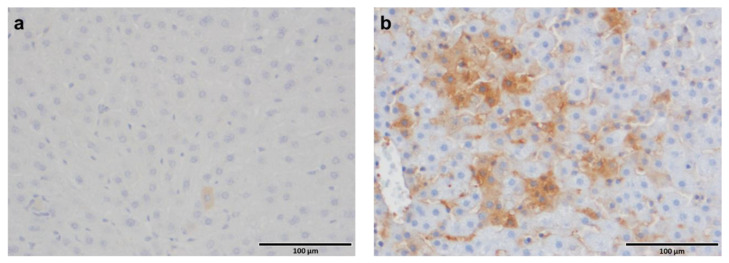
The presence of vi-VIM is strongly increased in the liver of WHV-infected woodchucks. Liver of one woodchuck (**a**) without WHV-infection and (**b**) with chronic WHV-infection was stained with hzVSF by IHC for the detection antibody-bound vi-VIM.

Overall, these results suggested that vi-VIM is strongly induced in vitro and in vivo by HBV in liver cells. Thus, liver tissues from WHV-uninfected and -infected woodchucks were stained with hzVSF by IHC for determining cross-reactivity of the humanized monoclonal antibody to woodchuck vi-VIM (Figure 6). Hepatocytes of WHV-uninfected woodchucks stained rarely positive for this protein, while hzVSF-bound vi-VIM was largely detected on liver cells of woodchucks with chronic WHV infection.

### 3.2. hzVSF Modestly Reduced Serum Viremia and Antigenemia in WHV-Infected Woodchucks, but the Antiviral Effect Was Enhanced in Combination with TAF and Durable in a Subset of Animals

After confirming the cross-reactivity of hzVSF to woodchuck vi-VIM, the safety of the humanized antibody was first assessed in a 12-week repeat-dose study in WHV-uninfected woodchucks at a dose of 16.0 mg/kg (Figure 1). Compared to placebo-treated controls, antibody treatment did not adversely affect body weights, body temperatures, hematology, and clinical chemistry parameters, and weights and histopathology of organs collected at necropsy (data not shown). Due to the absence of any safety concerns with the antibody, the therapeutic efficacy of repeat hzVSF administration at doses of 0.1, 4.0, and 16.0 mg/kg was subsequently evaluated for 12 weeks in WHV-infected woodchucks (Figure 1).

**Figure 7:**
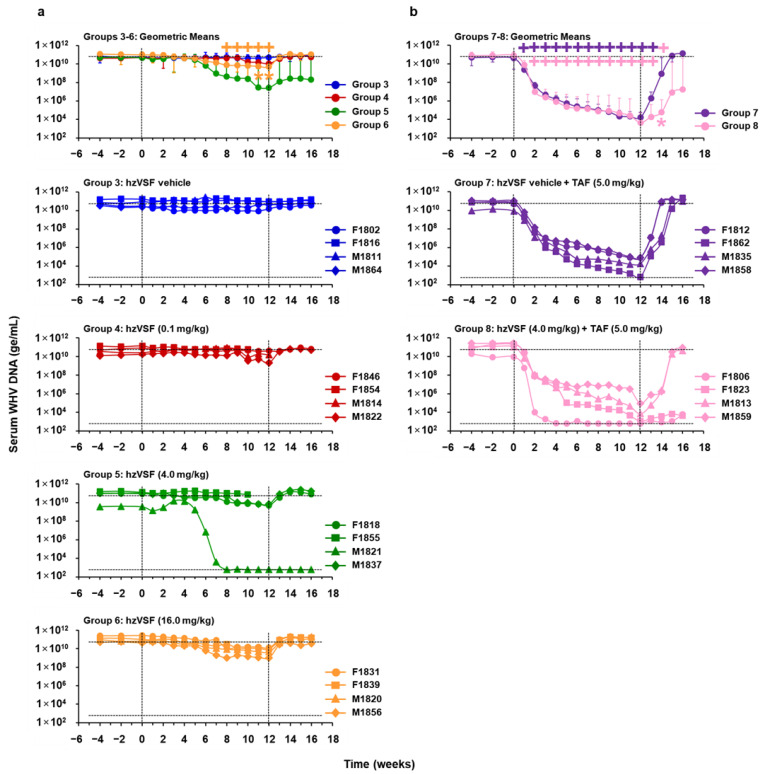
hzVSF monotreatment modestly reduces serum viremia, but suppression is enhanced in combination with TAF. Changes in serum WHV DNA levels relative to the pretreatment baseline (T0) of individual woodchucks treated with (**a**) placebo or hzVSF at a low, intermediate, and high dose and (**b**) TAF, alone or in combination with hzVSF at an intermediate dose. The top panels present the changes in the group geometric means of all mono and combination treatment regimens and the vertical lines indicate the standard error of the mean. The upper horizontal dotted lines present the mean viremia level of Groups 3–6 or Groups 7–8 at baseline, while the lower horizontal dotted lines indicate the detection limit for WHV DNA (i.e., 600 ge/mL). Vertical dotted lines at T0 and week 12 represent the duration of mono and combination treatment in this and the following figures. Viremia in Group 6 was significantly reduced compared to the baseline during weeks 8–12 (+: *p* < 0.05). At weeks 11 and 12, viremia in Group 6 was significantly reduced compared to Group 3 (*: *p* < 0.05). Compared to the baseline, viremia in Group 7 and Group 8 was significantly reduced during weeks 1–13 or during weeks 2–14, respectively (+: *p* < 0.05). At week 14, viremia in Group 8 was significantly reduced compared to Group 7 (*: *p* < 0.05). Abbreviation: ge/mL, genome equivalents or copy numbers per milliliter.

**Figure 8:**
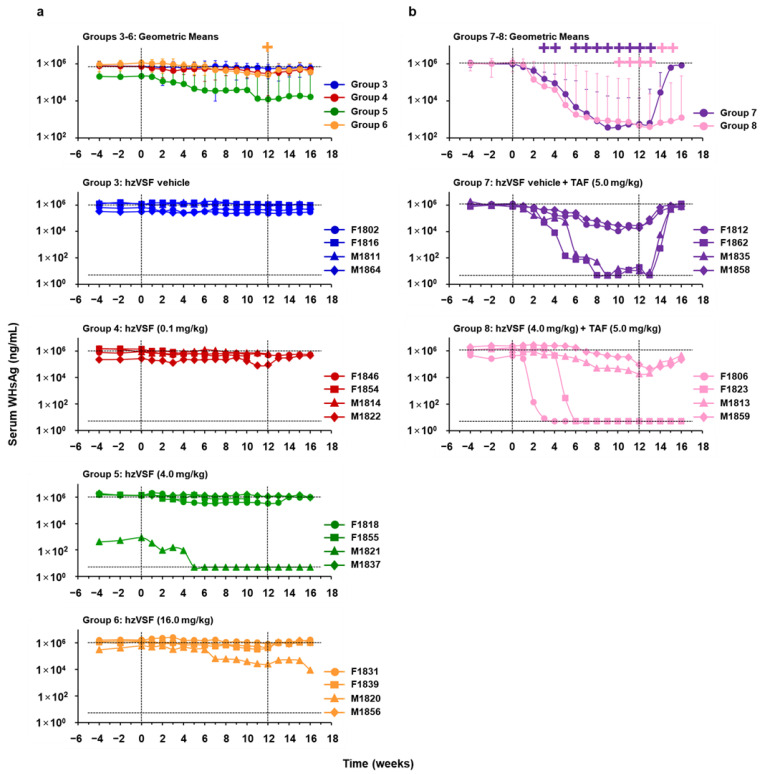
hzVSF monotreatment modestly reduces serum surface antigenemia, but suppression is enhanced in combination with TAF. Changes in serum WHsAg levels relative to the pretreatment baseline (T0) of individual woodchucks treated with (**a**) placebo or hzVSF at a low, intermediate, and high dose and (**b**) TAF, alone or in combination with hzVSF at an intermediate dose. The top panels present the changes in the group geometric means of all mono and combination treatment regimens and the vertical lines indicate the standard error of the mean. The upper horizontal dotted lines present the mean antigenemia level of Groups 3–6 or Groups 7–8 at baseline, while the lower horizontal dotted lines indicate the detection limit for WHsAg (i.e., 5 ng/mL). Antigenemia in Group 6 was significantly reduced compared to the baseline at week 12 (+: *p* < 0.05). Compared to the baseline, antigenemia in Group 7 and Group 8 was significantly reduced at weeks 3, 4, 6–13, and 15 or during weeks 10–15, respectively (+: *p* < 0.05).

As expected, placebo treatment of chronic WHV carrier woodchucks (Group 3) had no effect on serum viral markers (Figure 7a, Figure 8a and Figure S1a). Treatment of woodchucks with a low, intermediate, and high hzVSF dose reduced serum WHV DNA and WHsAg by 0.64, 3.22, and 1.29 log_10_ and 0.38, 1.24, and 0.90 log_10_ on average from the pretreatment baseline by the end of treatment (EOT) at week 12 in Group 4, Group 5, or Group 6, respectively. Serum WHeAg fluctuated in individual animals of these groups and on average increased by 0.47 ODU in Group 4, while it declined by 0.91 and 0.47 ODU at the EOT in Group 5 or Group 6, respectively. The more pronounced decline in mean viremia and antigenemia of Group 5 was due to the marked changes in one animal (M1821). Although only modest, the reductions in WHV DNA, WHsAg, and WHeAg from baseline in Group 6 were significant mainly at the EOT, and also significant in comparison to the control (Group 3). For assessing the durability of the treatment response, viral markers were measured for additional four weeks after treatment cessation. Viremia and antigenemia relapsed immediately in almost all animals and returned to or above baseline at the EOS at week 16. However, viral suppression was sustained in animal M1821, which presented with relatively low baseline viremia and antigenemia, and in which WHV DNA, WHsAg, and WHeAg became undetectable during treatment. Pharmacokinetic analysis indicated no differences in the hzVSF exposure of this and other animals in Group 5 or other groups (data not shown).

Based on the observation that the hzVSF-mediated antiviral effect was enhanced in one animal in the setting of low WHV replication (i.e., M1821 of Group 5), additional chronic WHV carrier woodchucks were treated with TAF for a rapid suppression of viral replication, alone and together with the antibody (Figure 1, Figure 7b, Figure 8b and Figure S1b). TAF treatment in Group 7 reduced serum WHV DNA, WHsAg, and WHeAg on average by 6.38 log_10_, 3.24 log_10_, or 1.86 ODU, respectively, at the EOT. The declines were significant during treatment, but the antiviral response was only transient, and viremia and antigenemia rebounded immediately in all animals after drug withdrawal. The addition of hzVSF at an intermediate dose to TAF treatment in Group 8 enhanced viral suppression, as serum WHV DNA and WHsAg declined on average by 7.27 log_10_ at the EOT or by 3.47 log_10_ at week 13, respectively, while the average WHeAg reduction of 1.87 ODU at week 13 was comparable to Group 7. In Group 8, WHV DNA became undetectable in one woodchuck (F1806) during treatment and WHsAg and WHeAg became absent in this and a second animal (F1823). After the cessation of combination treatment, WHV DNA stayed suppressed and WHsAg and WHeAg continued to be undetectable in both woodchucks, while viral markers relapsed in the other two animals of this group. The reductions in viremia and in surface and e antigenemia from baseline in Group 8 were significant during treatment, but also to Group 7 for WHV DNA and WHeAg after the EOT. This indicated that the relapse in these viral markers was slowed-down or delayed and that the magnitude of the rebound was reduced by combination treatment, due to two animals with durable WHV suppression. The relapse in WHsAg also appeared delayed, but the difference to Group 7 did not reach statistical significance.

### 3.3. hzVSF Minimally Reduced WHV Replication and Antigen Expression in the Liver of Woodchucks, but the Antiviral Effect Was Pronounced in Combination with TAF and Durable in a Subset of Animals

**Figure 9:**
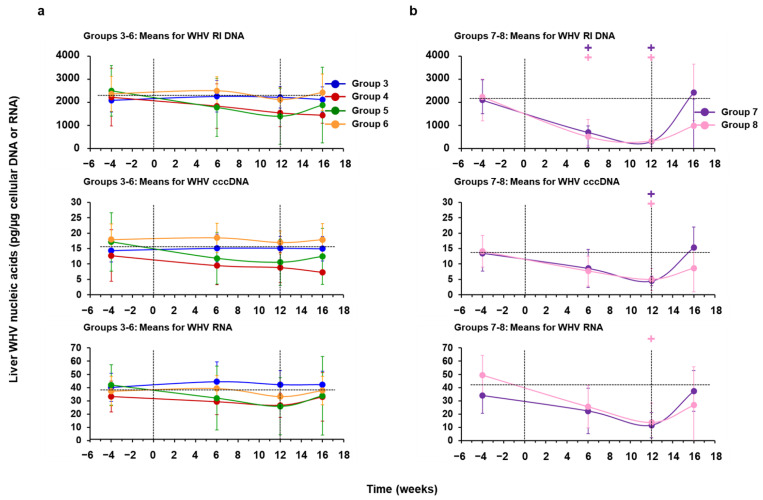
hzVSF minimally reduces WHV replication in the liver, but suppression is pronounced in combination with TAF. Comparison of means of (**a**) Groups 3–6 treated with placebo or hzVSF at a low, intermediate, and high dose and (**b**) Groups 7–8 treated with TAF, alone or in combination with hzVSF at an intermediate dose, relative to the pretreatment baseline (week −4) for intrahepatic levels of WHV DNA RI, cccDNA, and RNA. Vertical lines indicate the standard error of the mean. Horizontal dotted lines present the mean WHV nucleic acid levels of Groups 3–6 or Groups 7–8 at baseline. For the purpose of graphical presentation, hepatic WHV nucleic acids and antigens determined in terminal liver tissues after the EOS during weeks 17–19 are shown at week 16 in this and the following figure. WHV DNA RI and cccDNA in Groups 7 and 8 were significantly reduced compared to the baseline at weeks 6 and 12 or at week 12, respectively (+: *p* < 0.05). Compared to the baseline, WHV RNA in Group 8 was significantly reduced at week 12 (+: *p* < 0.05).

**Figure 10:**
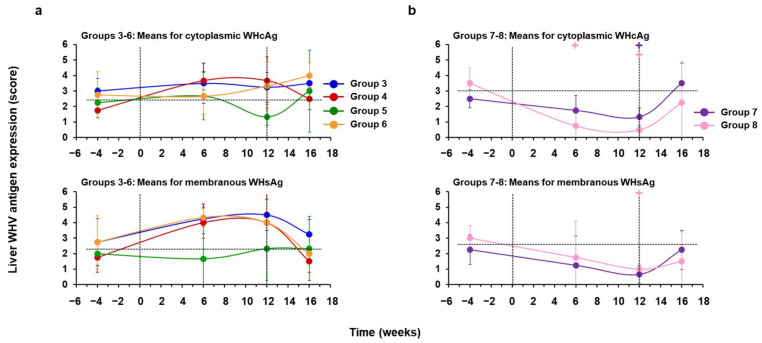
hzVSF does not reduce antigen expression in the liver, but suppression is pronounced in combination with TAF. Comparison of mean scores of (**a**) Groups 3–6 treated with placebo or hzVSF at a low, intermediate, and high dose and (**b**) Groups 7–8 treated with TAF, alone or in combination with hzVSF at an intermediate dose, relative to the pretreatment baseline (week −4) for cytoplasmic WHcAg and membranous WHsAg expression in liver. Vertical lines indicate the standard error of the mean. Horizontal dotted lines present the mean WHV antigen expression score of Groups 3–6 or Groups 7–8 at baseline. WHcAg in Group 7 and Group 8 was significantly reduced compared to the baseline at week 12 or at weeks 6 and 12, respectively (+: *p* <0.05). Compared to the baseline, WHsAg in Group 8 was significantly reduced at week 12 (+: *p* < 0.05). Score: 0 = 0%, 1 = 1–20%, 2 = 21–40%, 3 = 41–60%, 4 = 61–80%, and 5 = 81–100% of hepatocytes expressed WHcAg or WHsAg.

Placebo treatment of chronic WHV carrier woodchucks (Group 3) had no effect on viral DNA RI, cccDNA, and RNA, and on cytoplasmic WHcAg and membranous WHsAg expression in the liver (Figure 9a and Figure 10a, Figures S2a–S6a). In Group 4, Group 5, and Group 6, WHV DNA RI, cccDNA, and RNA were somewhat reduced by 0.13, 1.09, and 0.04 log_10_, by 0.07, 0.56, and 0.00 log_10_, or by 0.16, 0.48, and 0.04 log_10_, respectively, from the pretreatment baseline at the EOT. At the EOT, the score of WHcAg-stained hepatocytes was reduced only in Group 5 by 0.92, while declines in the score of WHsAg-stained hepatocytes were not noted. Thus, treatment of woodchucks with hzVSF mediated minimal effects on viral replication and antigen expression in the liver of most animals in Groups 4–6, and the average reduction was only minimal, always transient, appeared to be independent of the dose, and not statistically significant. The only exception was again animal M1821 of Group 5, with a pronounced and sustained viral suppression, as all WHV nucleic acids and proteins became undetectable during treatment and stayed absent until the EOS.

Treatment with TAF, alone (Group 7) and in combination with hzVSF (Group 8), reduced WHV DNA RI, cccDNA, and RNA by 0.87 and 2.14 log_10_, by 0.46 and 0.63 log_10_, or by 0.58 and 1.00 log_10_, respectively, from the pretreatment baseline at the EOT. At the EOT, the scores of WHcAg-and WHsAg-stained hepatocytes were reduced by 1.17 and 3.00 or by 1.58 and 2.00 in Group 7 or Group 8, respectively. Thus, TAF mono and combination treatment significantly reduced WHV DNA RI and cccDNA during treatment, while the decline in WHV RNA was also pronounced in both groups but only significant in Group 8 at the EOT (Figure 9b, Figures S2b–S4b). In addition, WHcAg expression was significantly reduced in Group 7 at the EOT, while WHcAg and WHsAg expression in Group 8 significantly declined during treatment or at the EOT, respectively (Figure 10b, Figures S5b and S6b). WHV suppression in Group 7 animals was transient, and viral nucleic acids and proteins in liver relapsed after drug withdrawal. This was different to the durable viral suppression in animals F1806 and F1823 of Group 8, with undetectable WHV RI DNA and RNA at the EOT and/or after the EOS. In addition, WHV cccDNA became undetectable in woodchuck F1806 during treatment. WHcAg and WHsAg expression also declined to undetectable levels in both animals during treatment and stayed absent thereafter. In the other two animals of this group, WHV nucleic acids and proteins declined during treatment but rebounded immediately after cessation of treatment.

### 3.4. hzVSF Treatment, Alone and in Combination with TAF, Elicited Antibodies to WHsAg and WHeAg in a Subset of Woodchucks

**Figure 11:**
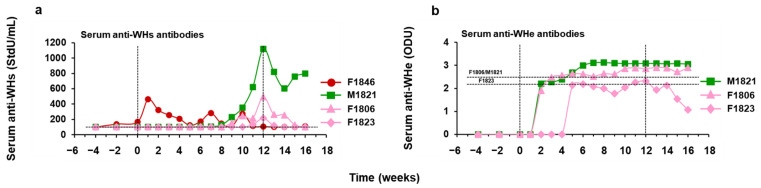
hzVSF treatment, alone and in combination with TAF, elicits antibodies to WHsAg and WHeAg in a subset of woodchucks. Changes in serum (**a**) anti-WHs antibody titers and (**b**) anti-WHe antibody levels in individual woodchucks during treatment with hzVSF at a low and intermediate dose (Groups 4–5) and TAF in combination with hzVSF at an intermediate dose (Group 8) relative to the pretreatment baseline (T0). The horizontal dotted lines indicate the detection limit for anti-WHs antibodies (i.e., 100 StdU/mL) or serum presence of anti-WHe antibodies (i.e., 2.52 ODU for F1806 and M1821 and 2.19 ODU for F1823). Abbreviations: StdU, standard unit; ODU, optical density unit.

During monotreatment with placebo (Group 3) or hzVSF at increasing doses (Groups 4–6), antibodies were typically undetectable in serum of chronic WHV carrier woodchucks (Figure 11). Woodchuck F1846 of Group 4 had a fluctuating anti-WHs antibody titer at pretreatment and during most of the study; however, anti-WHe antibodies were absent, consistent with the serum presence of WHeAg during the study. Animal M1821 of Group 5, with absent WHV replication at the EOS, developed durable anti-WHs and anti-WHe antibody responses during treatment. Woodchucks F1806 and F1823 of Group 8, with viral suppression at the EOS, elicited antibodies to both viral antigens during treatment, and the response was durable in the former animal, while it was transient in the latter animal.

### 3.5. Treatment with hzVSF, Alone or in Combination with TAF, Was Well Tolerated in Woodchucks

Comparable to WHV-uninfected woodchucks, body weights, body temperatures, hematology and clinical chemistry, and weights and histopathology of organs collected at necropsy of WHV-infected woodchucks were not adversely affected by treatment with hzVSF, alone and together with TAF (data not shown). This overall supported the safety of antibody and NA treatment in woodchucks with chronic WHV infection.

**Figure 12:**
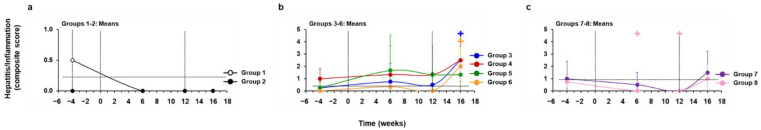
Treatment with hzVSF at the high dose or with TAF, alone and together with hzVSF at an intermediate dose, reduces inflammation in the liver. Comparison of mean composite scores of portal and sinusoidal hepatitis of (**a**) Groups 1–2 treated with placebo or hzVSF at a high dose, (**b**) Groups 3–6 treated with placebo or hzVSF at a low, intermediate, and high dose, and (**c**) Groups 7–8 treated with TAF, alone or in combination with hzVSF at an intermediate dose, relative to the pretreatment baseline (week −4). Vertical lines indicate the standard error of the mean. Horizontal dotted lines present the mean composite score of Groups 1–2, Groups 3–6, or Groups 7–8 at baseline. The composite score in Group 8 was significantly reduced compared to the baseline at weeks 6 and 12 *(*+: *p* < 0.05). Compared to the baseline, the composite score in Group 3 and Group 6 was significantly increased after the EOS (+: *p* < 0.05). The composite score was derived from the sinusoidal hepatitis score plus the portal hepatitis score. Composite score: 0 = absent, >0–2 = mild, >2–4 = moderate, and >4 = marked to severe hepatitis/liver inflammation.

Further supporting the safety of hzVSF, changes in portal and sinusoidal hepatitis were absent in almost all WHV-uninfected woodchucks of Groups 1–2, except for one placebo-treated animal (M1747) with a reduction in the composite score at week 6 (Figure 12, Figure S7). WHV-infected woodchucks of Group 3 administered placebo and of Groups 4–5 treated with a low and intermediate hzVSF dose had (transient) increases in liver inflammation during treatment and after the EOS. This was in contrast to animals of Group 6 treated with the high hzVSF dose, Group 7 treated with TAF, and Group 8 treated with TAF and hzVSF at a medium dose, which showed declines in liver inflammation during treatment at weeks 6 and/or 12. The reduction in the composite score from the pretreatment baseline, however, was only significant in Group 8 during treatment. After the cessation of treatment, most animals in Groups 6–8 experienced an increase in liver inflammation and the composite scores were often higher after the EOS than those at baseline. Liver inflammation was varied in woodchucks with absent or suppressed viral replication after the EOS, as portal and sinusoidal hepatitis stayed absent in animals M1821 (Group 5) and F1823 (Group 8), while both disease markers increased in animal F1806 (Group 8).

**Figure 13:**
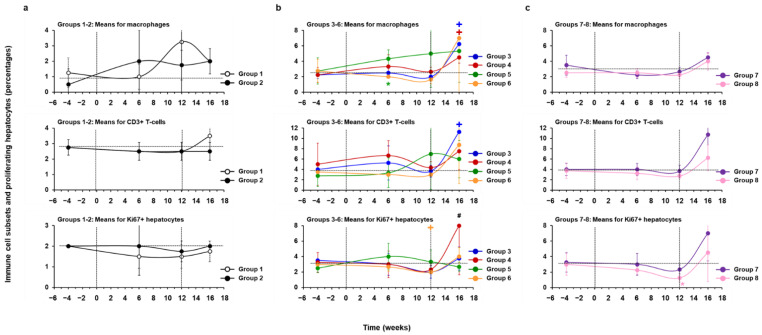
Treatment with hzVSF at the high dose or with TAF, alone and together with hzVSF at an intermediate dose, reduces the presence of intrahepatic immune cell subsets and lowers liver replenishment by new hepatocytes. Comparison of mean percentages of macrophages, CD3+ T-cells, and Ki67+ hepatocytes of (**a**) Groups 1–2 treated with placebo or hzVSF at a high dose, (**b**) Groups 3–6 treated with placebo or hzVSF at a low, intermediate, and high dose, and (**c**) Groups 7–8 treated with TAF, alone or in combination with hzVSF at an intermediate dose, relative to the pretreatment baseline (week –4). Vertical lines indicate the standard error of the mean. Horizontal dotted lines present the mean percentage of Groups 1–2, Groups 3–6, or Groups 7–8 at baseline. Macrophages in Group 3 and Group 4 were significantly increased compared to the baseline after the EOS during weeks 17–19 (+: *p* < 0.05). Compared to Group 3 and Group 6, macrophages were significantly increased in Group 5 at week 6 (*: *p* < 0.05). CD3+ T-cells in Group 3 were significantly increased compared to the baseline after the EOS during weeks 17-19 (+: *p* < 0.05). Ki67+ hepatocytes in Group 6 were significantly reduced compared to the baseline at week 12 (+: *p* < 0.05). Compared to Group 7, Ki67+ hepatocytes were significantly reduced in Group 8 at week 12 (*: *p* < 0.05). ^#^ The mean Ki67+ hepatocyte percentage in Group 4 was 15.5%.

In alignment with the reduced liver inflammation during treatment, the numbers of macrophages and CD3+ T-cells within liver of woodchucks in Groups 6–8 were largely unchanged or even declined from the pretreatment baseline, but markedly increased after treatment cessation (Figure 13, Figures S8 and S9). This in turn was associated with a lower liver replenishment, as the numbers of proliferating, Ki67+ hepatocytes transiently declined during treatment (Figures S10 and S13). The reduction in the mean percentage of proliferating hepatocytes from the pretreatment baseline was significant in Group 6 at the EOT. In addition, the decline in proliferating hepatocytes in Group 8 was significant to Group 7 at the EOT. Cell subset changes in woodchucks with absent or suppressed viral replication after the EOS were comparable during treatment and more varied after drug withdrawal. Macrophages, CD3+ T-cells, and Ki67+ hepatocytes declined or remained unchanged during treatment, but continued to stay unaltered in animal M1821 (Group 5) or increased slightly after drug withdrawal in animals F1806 and F1823 (Group 8), with a more pronounced increase in proliferating hepatocytes in the former animal after the EOS. The above reductions in cell subsets were different to woodchucks of Groups 3–5, in which macrophages and CD3+ T-cells often (transiently) accumulated in the liver during treatment or at the EOT, while Ki67+ hepatocytes stayed nearly unchanged or were transiently increased during treatment. Cell numbers increased further after the cessation of treatment, except for CD3+ T-cells and proliferating hepatocytes in Group 5. WHV-uninfected woodchucks of Group 2 treated with hzVSF had nearly unchanged numbers of CD3+ T-cells and Ki67+ hepatocytes during treatment, while macrophages increased in parallel. This was in contrast to placebo-treated animals of Group 1, in which macrophages and CD3+ T-cells increased during treatment or after the EOS, respectively, while Ki67+ hepatocytes somewhat declined during the study.

**Figure 14:**
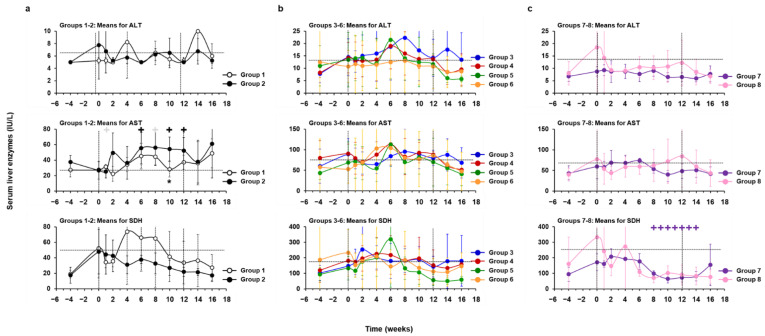
Treatment with hzVSF at the high dose or at an intermediate dose in combination with TAF does not significantly elevate serum liver enzymes. Comparison of mean levels of ALT, AST, and SDH of (**a**) Groups 1–2 treated with placebo or hzVSF at a high dose, (**b**) Groups 3–6 treated with placebo or hzVSF at a low, intermediate, and high dose, and (**c**) Groups 7–8 treated with TAF, alone or in combination with hzVSF at an intermediate dose, relative to the pretreatment baseline (T0). Vertical lines indicate the standard error of the mean. Horizontal dotted lines present the mean levels of Groups 1–2, Groups 3–6, or Groups 7–8 at baseline. The mean AST levels in Group 1 and Group 2 were significantly increased compared to the baseline at weeks 1 and 8 or at weeks 6, 10, and 12, respectively (+: *p* < 0.05). Compared to Group 1, the mean AST level was significantly increased in Group 2 at week 10 (*: *p* < 0.05). The mean SDH level in Group 7 was significantly reduced compared to the baseline during weeks 8–14 (+: *p* < 0.05). Compared to Group 4, the mean SDH level was significantly reduced in Group 5 at week 16 (*p* < 0.05). Abbreviation: IU, international unit.

Correlating further with the reduced liver inflammation during treatment, the mean serum levels of the liver enzymes ALT, AST, and SDH were not significantly elevated from the pretreatment baseline in most groups during the study (Figure 14 and Figures S11–S13). The only exception was a significantly higher AST level in WHV-uninfected woodchucks of Group 2 during hzVSF treatment at week 10, when compared to placebo-treated animals of Group 1. Individual WHV-uninfected and -infected woodchucks across the experimental groups sometimes presented with pronounced elevations in liver enzymes; however, these increases were sporadically observed at pretreatment, during treatment, and/or during the follow-up. Most often, the same animals within a group had elevations in two or all three liver enzymes around the same time, and changes in ALT and AST levels were not limited to animals with suppressed or absent WHV replication after the EOS. SDH elevations were typically present in woodchucks with more pronounced declines in serum viremia and/or antigenemia during treatment with hzVSF, alone and together with TAF, such as animals M1822 (Group 4), M1821 and F1855 (Group 5), M1856 (Group 6), and F1806 and F1823 (Group 8), suggesting an effect mediated by the monoclonal antibody. This was opposite to TAF monotreatment in Group 7 animals, with no pronounced changes in SDH levels, suggesting absent or only minor effects by the NA. Nevertheless, liver enzyme increases in individual woodchucks reversed, and AST levels in Group 7 normalized during treatment, as also noted for SDH in Group 8.

## 4. Discussion

In the present study, the humanized antibody hzVSF was shown to bind to vi-VIM induced by HBV and WHV in liver cells following infection of humans or woodchucks, respectively. Increased protein presence at the cell surface correlated further with the progressing liver disease, and much more so in HBV-infected patients with CHB and cirrhosis than in patients with HBV-unrelated liver sequelae. VIM expression during cancer development has been found to correlate with tumor growth, and the increased presence of surface VIM on tumor endothelial cells may indicate a role of this protein in tumor metastasis and/or invasion [61,62]. Thus, the upregulation of VIM at the cell surface is contingent on the health status of the liver and may not be entirely depend on viral infection. Furthermore, the presence of surface VIM/vi-VIM is not restricted to infection with hepadnaviruses, as other viruses, such as human severe acute respiratory syndrome-related coronavirus (SARS-CoV), Japanese encephalitis virus (JEV), and enterovirus 71 (EV-A71), also induce this filamentous protein at least in vitro [23,63,64,65]. Surface VIM/vi-VIM is further used by SARS-CoV, JEV, EV-A71, porcine reproductive and respiratory syndrome virus, dengue virus, H9N2 subtype avian influenza virus, Chandipura virus, and cowpea mosaic virus as a receptor or at least as a co-receptor for cell attachment and entry by endocytosis, and in many studies viral infection can be reduced or even inhibited in vitro by using antibodies against VIM presented at the cell surface [62,64,66,67,68,69,70,71]. Although the exact mechanism of surface VIM/vi-VIM induction needs to be elucidated, infection with rather diverse viruses apparently changes the conformation of intracellular VIM thereby allowing its presentation on the cell surface. The pronounced increase and subsequent decline in intracellular VIM shortly after HBV infection of human hepatoma cells in the present study supports the proposed conformational change and further revealed a perinuclear colocalization of both VIM proteins, although vi-VIM was partially more condensed and formed filamentous structures radiating to the cell periphery. This observation, together with the reported involvement of VIM in receptor ligand transendocytosis [36], may suggest that vi-VIM participates in the clathrin- and/or EGFR-mediated endocytosis of NTCP-bound HBV and/or subsequent endosome maturation by translocating early endosomes containing the HBV receptor complex from the cell periphery to a more perinuclear region. Consequently, targeting vi-VIM on human hepatoma cells with hzVSF inhibited NTCP receptor-mediated endocytosis of HBV by likely altering the intracellular VIM localization [35]. This study concluded that hzVSF acts as an HBV entry inhibitor and that antibody treatment mainly prevents the reinfection of already infected hepatocytes. Thus, the proposed mechanism of action of hzVSF is different to Myrcludex B, a synthetic peptide homologous to the preS1 domain of the HBV L protein that interferes with the binding of HBV virions at the NTCP receptor and that primarily inhibits the de novo infection of uninfected hepatocytes, as postulated from studies in the humanized mouse model of HBV and in patients with CHB [22,72]. The antiviral activity of hzVSF appears more comparable to compounds that inhibit HBV endocytosis in cell culture. For example, ponesimod was shown to suppress the endosomal maturation of HBV virions by inhibiting the conversion of early endosomes to late endosomes, causing HBV particles to be retained inside the early endosomes and thus to prevent nucleocapsid dissociation and release into the cytoplasm [73]. Two other compounds, silibinin and pigallocatechin-3-gallate, were reported to block HBV entry by either hindering clathrin-mediated endocytosis of the HBV/NTCP receptor complex [74] or by inducing clathrin-dependent endocytosis of HBV-free NTCP from the plasma membrane resulting in receptor degradation [75].

Following confirming the cross-reactivity of hzVSF to woodchuck vi-VIM in the liver, the safety and therapeutic efficacy of the humanized antibody was evaluated in this preclinical animal model of HBV. Repeat iv administration of hzVSF was safe in WHV-uninfected and -infected woodchucks, as, among several other parameters tested (i.e., body weight and temperature, serum chemistry, and hematology), gross, weight, and histopathology changes in the liver and other organs were absent that are indicative of an anti-drug antibody reaction against hzVSF. Different to Myrcludex B treatment for 24 weeks [22], it seems unlikely that patients treated repeatedly with the humanized antibody during future clinical trials will develop antibodies against hzVSF thereby neutralizing the effect of this compound and possibly affecting its overall safety profile. Since the direct target of hzVSF is vi-VIM instead of NTCP, changes in the transport of conjugated bile acids by the receptor are not expected, as reported for Myrcludex B [22]. Comparable to Myrcludex B, which reduced the serum HBV load by more than 1.0 log_10_ in two out of eight HBe-negative patients with HBV/HDV coinfection after 24 weeks (average reduction 0.29 log_10_) [22], hzVSF monotreatment of WHe-positive woodchucks produced a modest albeit significant average decline of 0.90 log_10_ in WHV DNA level after 12 weeks at the highest dose. While Myrcludex B did not affect HBsAg levels in patients, the highest dose of hzVSF modestly but significantly reduced the levels of WHsAg and WHeAg. These results in woodchucks are in agreement with the proposed mechanism of action of hzVSF insofar that blocking the continual reinfection of already WHV-infected hepatocytes can only facilitate a minor effect on the maintenance of the chronic infection by modulating the replenishment of the cccDNA pool in infected hepatocytes. Prolonged hzVSF treatment of chronic WHV carrier woodchucks most likely interfered with the generation of cccDNA from rcDNA of incoming extracellular virions. The cccDNA molecule serves as the template for transcription of all viral RNAs, including the pre-genomic (pg) RNA which is reverse transcribed into rcDNA in newly formed nucleocapsids and then secreted within new virions or recycled back to the nucleus [5]. Continued blocking of virion entry into hepatocytes while new virions constantly egress from these cells may further support lowering the cccDNA pool via reduced intracellular amplification from rcDNA in available cytoplasmic nucleocapsids. However, hzVSF treatment produced only minor effects on WHV DNA RI, cccDNA, and RNA in the liver that appeared to be independent of the applied dose, but the declines in these viral molecules were sometimes more evident in individual animals. Contrary to the surface and e antigenemia levels in the periphery, core and surface expression in the liver remained unaffected by hzVSF monotreatment. This discrepancy in peripheral and intrahepatic marker changes may be explainable by the different sensitivity of the used assays (i.e., PCR, ELISA, and IHC). In addition, reductions in intrahepatic WHV DNA RI, cccDNA, and RNA were determined by Southern/Northern blot assays that are less sensitive than PCR-based assays. Overall, hzVSF-mediated entry inhibition appeared not to directly interfere with late steps of WHV replication and the modest effects on the secretion of WHV virions, subviral particles, and e antigen likely related to the minor effect of hzVSF on cccDNA that allowed continued but somewhat lower RNA transcription. Since withdrawal of hzVSF monotreatment led to an immediate viral relapse, as also noted for Myrcludex B when used as a single agent in the humanized mouse model of HBV [72], this indicated that the induced antiviral effect depends on the continued presence of the antibody or peptide. Although the hzVSF-mediated treatment response in the periphery was dose-dependent, it is unknown if the highest antibody dose administered to woodchucks was able to bind to all vi-VIM presented initially on the surface of hepatocytes. Thus, future studies should attempt to define an optimal antibody dose for binding all vi-VIM in the liver and to eventually extend the treatment duration for enhancing the antiviral efficacy of hzVSF, for example to 24 weeks in analogy to treatment of patients with Myrcludex B [22]. The treatment duration, however, will depend on several factors, including the half-life time of cccDNA, the natural turnover rate of infected cells, the replenishment of the liver with new, uninfected hepatocytes, and the underlying immune response attempting to control the persistent infection by removal of infected hepatocytes. Accordingly, combination of entry inhibition with compounds targeting other steps of the viral life cycle and affecting the extracellular and intracellular replenishment of the cccDNA pool may act synergistically. Combinational treatment with a NA was selected in the current study following the observation that a durable antiviral effect was rather rapidly induced with hzVSF in one woodchuck with low basal levels of WHV replication, including intrahepatic cccDNA load.

The above discussed modulation of chronic WHV infection by hzVSF monotreatment was clearly different to conventional NA therapy, as investigated for the first time with TAF in woodchucks of the present study. TAF is a prodrug of tenofovir with greater plasma stability and uptake by hepatocytes than tenofovir disoproxil fumarate (TDF) [53]. As a nucleotide analog, tenofovir potently inhibits the HBV DNA polymerase and leads to a suppression of HBV replication in patients with CHB in regard to viremia, but usually does not significantly reduce antigenemia [53,76]. Contrary to adverse renal events and bone loss sometimes associated with TDF treatment [77], TAF is safer and antiviral efficacious at an approximately 10-fold lower dose. Without performing an initial dose-finding study, the applied TAF dosage (i.e., 5 mg/kg) reduced rather uniformly the WHV DNA serum load in all woodchucks, while the effect on circulating e and surface antigen levels was greatly varied in individual animals. This is consistent to treatment with other NAs in woodchucks, including entecavir and TDF, during prolonged administration at relatively high doses [47,54,78,79,80]. Consistent with these studies, TAF also reduced intrahepatic WHV DNA RI, cccDNA, and RNA to varying degrees in woodchucks. Since the antiviral effect produced by TAF in the current study (and by ETV and TDF in comparable studies) was always transient, and immediate reoccurrence of WHV replication was noted in all woodchucks after treatment cessation, this indicated that the decline in viral markers is dependent on the continued presence of NAs. However, when TAF was provided together with hzVSF, the antiviral effect was enhanced, and a more profound suppression of WHV markers in serum and liver was achieved with the combination treatment regimen than with either drug alone. In addition, the treatment response was durable in two woodchucks receiving TAF plus hzVSF, based on seroconversion and suppressed or undetectable WHV replication during the follow-up period of 5–7 weeks. Since the TAF-induced antiviral effect was always transient, this emphasizes the significance of the sustained antiviral response mediated by combination treatment in half of the animals. The antiviral effects here were comparable to those in the one animal with low viral replication markers at the initiation of hzVSF monotreatment, and likewise both woodchucks had (somewhat) lower levels of WHV cccDNA at the initiation of hzVSF/TAF combination treatment. The underlying mechanism for the superior response to combination treatment may involve, in addition to the development of antiviral B- and T-cell responses (see below), a reduced number of virions in the circulation by TAF treatment and an efficient blocking of these remaining virions from hepatocyte reinfection by hzVSF administration, which significantly affected the cccDNA pool and RNA transcription in these animals. For reasons not understood yet, the antiviral response in the other half of woodchucks undergoing combination treatment was transient, and thus comparable to all other animals after cessation of monotreatment with either hzVSF or TAF. Overall, these results indicated a variability in the individual responsiveness to hzVSF that may depend on the levels of WHV cccDNA at treatment initiation, but primarily show that the durability of the treatment response was dependent on markedly reduced levels of WHV replication, either naturally attained by the underlying immune response (in the absence of testing for the presence of an unlikely WHV genome mutation) or induced by TAF. This is comparable to other woodchuck studies in which agonism of toll-like receptors 7 and 9 in combination with entecavir produced superior effects over monotreatment with each drug, albeit only in subsets of animals [56,78,81]. A more pronounced effect of Myrcludex B on HBV DNA serum loads when provided in combination with PegIFN-α2a to HBV/HDV co-infected patients has been also observed [22]. The noted variability in the antiviral response to hzVSF mono and combination treatment is not unexpected considering the outbreed nature of woodchucks and the unknown severity of CHB and associated impairment of antiviral immunity at treatment initiation in individual animals.

Antibodies to WHsAg (and WHeAg) were not elicited in almost all woodchucks of the current study and may additionally explain the immediate viral relapse observed after cessation of monotreatment with hzVSF or TAF, and in part after withdrawal of combination treatment. Although viral surface and e antigen levels transiently declined during mono and combination treatment in the present and in other woodchuck studies, the magnitude and/or duration of antigenemia suppression apparently was insufficient to remove both tolerizing antigens from the system for a retrieval of the antibody-producing functions of plasma B-cells, as also described for patients with CHB [82,83,84]. However, in the three woodchucks with suppressed or undetectable WHV replication mediated by hzVSF, alone and in combination with TAF, the declines in antigenemia were sufficient for the restoration of these B-cell functions and allowed the elicitation of anti-WHV antibodies, as also reported for patients with NA treatment-induced seroconversion [84]. This finding in turn rules out the possibility that these antibodies were undetectable in other treated animals because of their presence in complexes with WHsAg or WHeAg. This is supported by several treatment studies in woodchucks with compounds other than NAs that increased the rate of seroconversion. These compounds indirectly or directly activated B-cells, such as toll-like receptor 7, 8, and 9 agonists [49,56,78,85], or treatment regimens were applied involving a combination of NA and therapeutic vaccine [42,86], in addition to blockage of checkpoint inhibitors [79], for the induction of a humoral response. As WHV-specific cellular responses were not assessed in the present study, it is unknown if hzVSF treatment, alone and together with TAF, also retrieved the antiviral functions of T helper cells and/or cytolytic T lymphocytes (CTLs), and if these cell subsets contributed to the declines in viremia and antigenemia. A clear relationship between elevations in the liver enzymes ALT and AST and a sustained treatment response was not apparent. SDH, another liver enzyme used as a marker of liver cell injury in woodchucks, transiently increased in animals with more noticeable hzVSF-induced declines in WHV replication, and thus may indicate an attempted immune control of WHV by a cytolytic mechanism, as also reported for HBV in patients [87]. However, removal of WHV-infected hepatocytes by CTLs did not appear to be the main antiviral activity of hzVSF, because SDH levels normalized and liver inflammation declined during monotreatment, and even more so in combination with TAF. The changes in SDH level in woodchucks are comparable to the declines and normalization of ALT levels in six of eight HBV/HDV co-infected patients during treatment with Myrcludex B for 24 weeks [22]. As intrahepatic CD3+ T-cell accumulation and liver replenishment with new (i.e., proliferating) hepatocytes for eventual compensation of cell loss also decreased in woodchucks during treatment, this pointed more to WHV entry inhibition than cytolytic viral control by hzVSF as the underlying antiviral mechanism, in addition to the induced humoral response. Since macrophage infiltration in the liver of treated woodchucks was also reduced, these results are in agreement with the reported anti-inflammatory properties of hzVSF in regard to treatment of severe COVID-19 symptoms that led to a marked decline in the serum levels of C-reactive protein, interleukin 6, tumor necrosis factor-α, and CC chemokine ligand-2 or monocyte chemoattractant protein 1 [37]. Of note is that VIM deficiency in mouse-based disease models reduces inflammation [33], including absent inflammasome activation in the brain after EV-A71 infection [88] and enhanced production of reactive oxygen species by activated macrophages in the gut after *Escherichia coli* infection [89]. Furthermore, antibody-mediated blocking of surface VIM presented by a subset of activated peripheral blood T-cells inhibits their proliferation and cytokine secretion [90,91].

In conclusion, the present study in the woodchuck model of HBV demonstrates the potential of combining the novel, humanized antibody hzVSF with a NA for achieving sustained suppression of chronic hepadnavirus infection. After binding to HBV-induced VIM presented at the surface of infected liver cells, hzVSF inhibits viral cell entry via NTCP receptor-based endocytosis of virions [35]. In woodchucks, the hzVSF-mediated entry inhibition modestly changed the course of chronic WHV infection, but the antiviral activity of the antibody was augmented by providing additional and marked viral suppression via TAF treatment. The combination regimen further reduced the levels of tolerizing viral antigens and reversed the impaired B-cell functions in at least a subset of woodchucks. By analogy, these results suggest that hzVSF treatment on top of standard of care with NAs (and possibly in combination with PegIFN-α as well) may safely and durably suppress chronic HBV infection in patients, and thus presents a new therapeutic option to increase the low HBV cure rate currently achieved with available drugs. Although it has not been investigated yet, it is conceivable that hzVSF is also antiviral efficacious against HDV, as this satellite virus is using the HBV envelope for attachment to and endocytosis with the NTCP receptor [10].

## Data Availability

All relevant data generated during the study are presented within the manuscript and the Supplementary Materials.

## Supplementary Materials

The following are available online at https://www.mdpi.com/article/10.3390/cells10092321/s1, Table S1: Allocation of woodchucks to treatment groups, Figure S1: hzVSF monotreatment modestly reduces serum e antigenemia, but suppression is enhanced in combination with TAF, Figure S2: hzVSF minimally reduces WHV DNA RI in the liver, but suppression is pronounced in combination with TAF, Figure S3: hzVSF minimally reduces WHV cccDNA in the liver, but suppression is pronounced in combination with TAF, Figure S4: hzVSF minimally reduces WHV RNA in the liver, but suppression is pronounced in combination with TAF, Figure S5: hzVSF does not reduce cytoplasmic WHcAg expression in the liver, but suppression is pronounced in combination with TAF, Figure S6: hzVSF does not reduce membranous WHsAg expression in the liver, but suppression is pronounced in combination with TAF, Figure S7: Treatment with hzVSF at the high dose or with TAF, alone and together with hzVSF at an intermediate dose, reduces inflammation in the liver, Figure S8: Treatment with hzVSF at the high dose or with TAF, alone and together with hzVSF at an intermediate dose, reduces the number of macrophages within liver, Figure S9: Treatment with hzVSF at the high dose or with TAF, alone and together with hzVSF at an intermediate dose, reduces the number of CD3+ T-cells within liver, Figure S10: Treatment with hzVSF at the high dose or with TAF, alone and together with hzVSF at an intermediate dose, lowers liver replenishment by new hepatocytes, Figure S11: Treatment with hzVSF at the high dose or at an intermediate dose in combination with TAF does not induce elevations in serum ALT in most woodchucks, Figure S12: Treatment with hzVSF at the high dose or at an intermediate dose in combination with TAF does not induce elevations in serum AST in most woodchucks, Figure S13: Treatment with hzVSF at the high dose or at an intermediate dose in combination with TAF does not induce elevations in serum SDH in most woodchucks.
