# Supplementary material for: Combination Treatment with the Vimentin-Targeting Antibody hzVSF and Tenofovir Suppresses Woodchuck Hepatitis Virus Infection in Woodchucks
Source: Cells. 2021 Sep 5;10(9):2321. doi: 10.3390/cells10092321 (PMC8466705; doi:10.3390/cells10092321)
Supplement: Supplementary file 1 [file cells-10-02321-s001.zip › cells-1323245-supplementary.pdf]

Supplementary Data

Table S1. Allocation of woodchucks to treatment groups.

Study	Group	WHV Status	Treatment	N	Animal Identification
1	1	Negative	hzVSF vehicle	4	F1702, F1706, M1733, M1747
	2	Negative	hzVSF (16.0 mg/kg)	4	F1716, F1731, M1722, M1734
2	3	Positive	hzVSF vehicle	4	F1802, F1816, M1811, M1864
	4	Positive	hzVSF (0.1 mg/kg)	4	F1846, F1854, M1814, M1822
	5	Positive	hzVSF (4.0 mg/kg)	4	F1818, F1855, M1821, M1837
	6	Positive	hzVSF (16.0 mg/kg)	4	F1831, F1839, M1820, M1856
3	7	Positive	hzVSF vehicle + TAF (5.0 mg/kg)	4	F1812, F1862, M1835, M1858
	8	Positive	hzVSF (4.0 mg/kg) + TAF (5.0 mg/kg)	4	F1806, F1823, M1813, M1859

N, number per group; M, male; F, female.

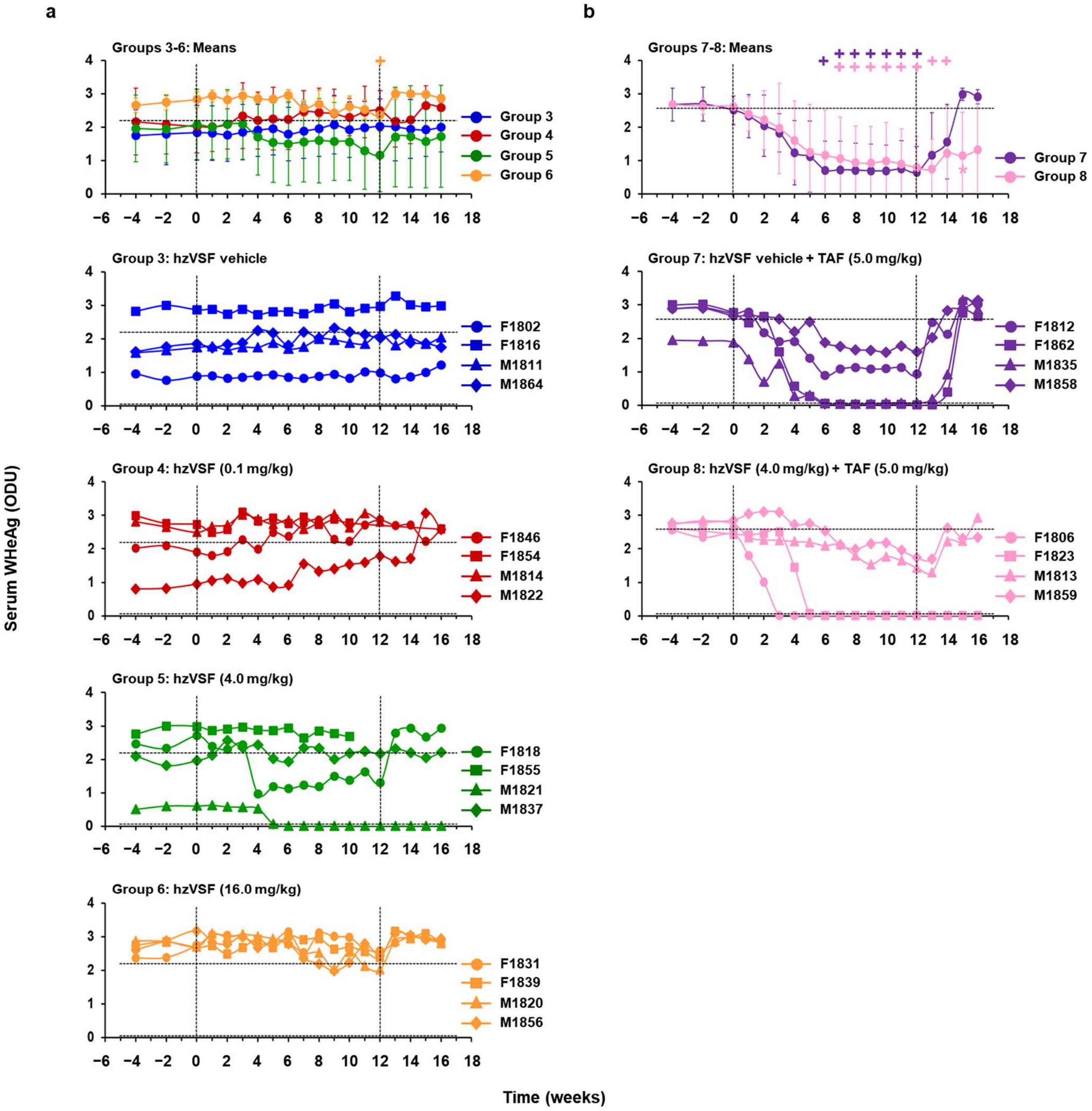

Figure S1. hzVSF monotreatment modestly reduces serum e antigenemia, but suppression is enhanced in combination with TAF. Changes in serum WHeAg levels relative to the pretreatment baseline (T0) of individual woodchucks treated with (**a**) placebo or hzVSF at a low, intermediate, and high dose and (**b**) TAF, alone or in combination with hzVSF at an intermediate dose. The top panels present the changes in the group means of all mono and combination treatment regimens and the vertical lines indicate the standard error of the mean. The upper horizontal dotted lines present the mean antigenemia level of Groups 3–6 or Groups 7–8 at baseline, while the lower horizontal dotted lines indicate the detection limit for WHeAg (i.e., 0.056 ODU). Vertical dotted lines at T0 and week 12 represent the duration of mono and combination treatment in this and following figures. Antigenemia in Group 6 was significantly reduced

compared to the baseline at week 12 (+: $p < 0.05$). Compared to the baseline, antigenemia in Group 7 and Group 8 was significantly reduced during weeks 6–12 or weeks 7–14, respectively ($^{+}$: $p < 0.05$). At week 15, antigenemia in Group 8 was significantly reduced compared to Group 7 (*: $p < 0.05$). Abbreviation: ODU, optical density unit.

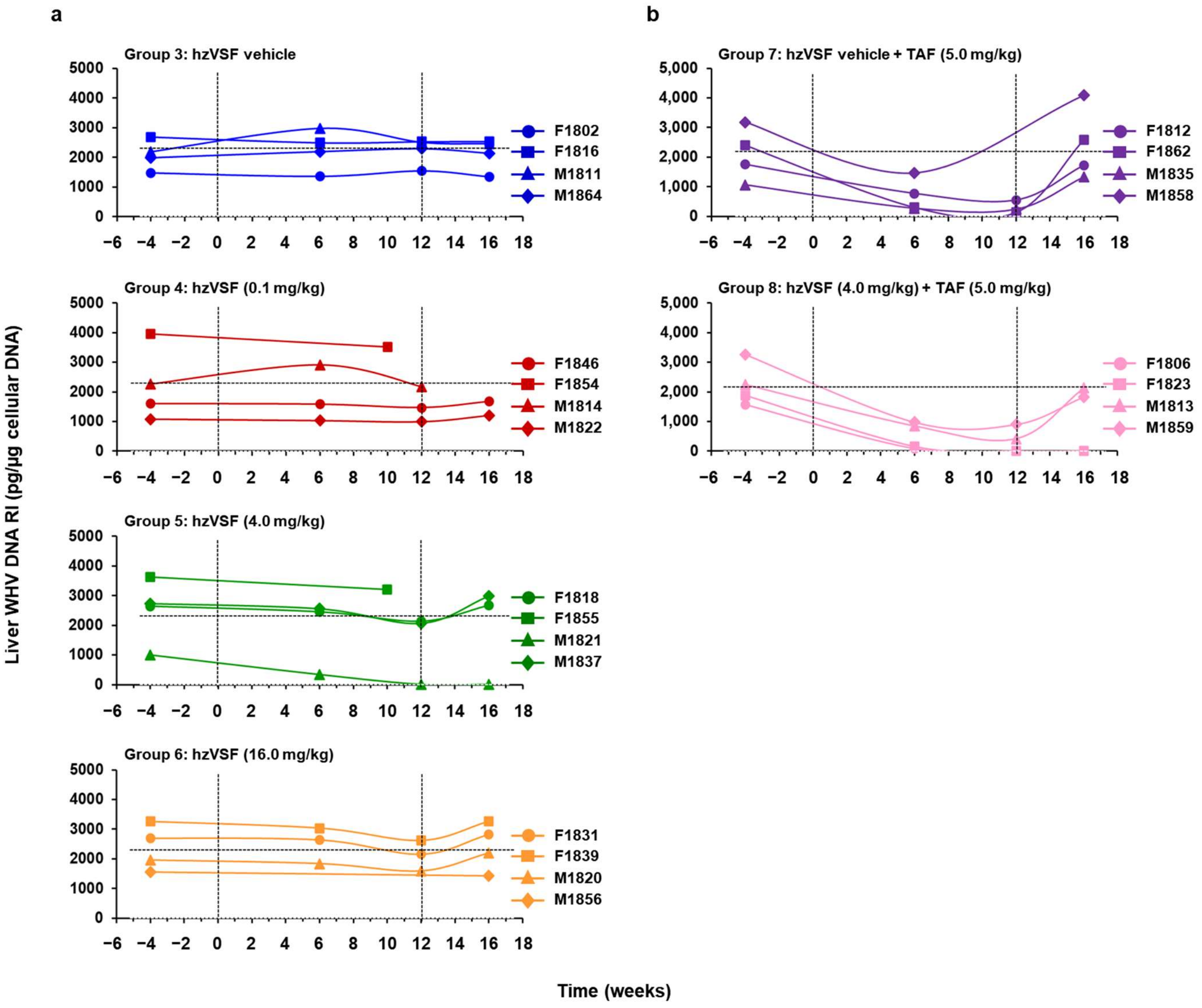

Figure S2. hzVSF minimally reduces WHV DNA RI in the liver, but suppression is pronounced in combination with TAF. Changes in intrahepatic WHV DNA RI levels relative to the pretreatment baseline (week −4) of individual woodchucks treated with (**a**) placebo or hzVSF at a low, intermediate, and high dose and (**b**) TAF, alone or in combination with hzVSF at an intermediate dose. The upper horizontal dotted lines represent the mean WHV DNA RI level of Groups 3–6 or Groups 7–8 at baseline, while the lower horizontal dotted lines indicate the detection limit for this molecule (2.0 pg/µg cellular DNA). For the purpose of graphical presentation, WHV nucleic acids and antigens determined in terminal liver tissues after the EOS during weeks 17-19 are shown at week 16 in this and the following figures.

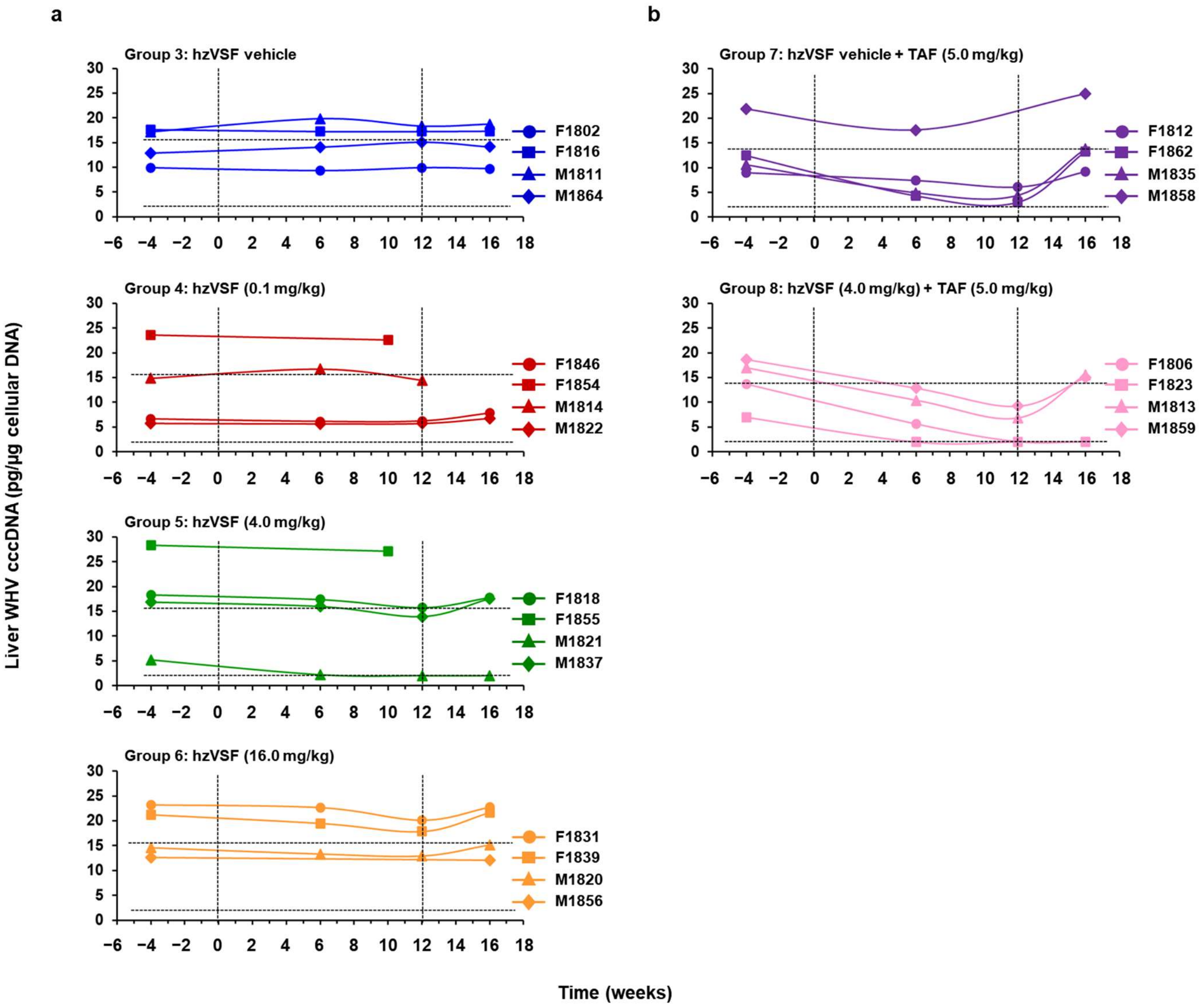

Figure S3. hzVSF minimally reduces WHV cccDNA in the liver, but suppression is pronounced in combination with TAF. Changes in intrahepatic WHV cccDNA level relative to the pretreatment baseline (week −4) of individual woodchucks treated with (**a**) placebo or hzVSF at a low, intermediate, and high dose and (**b**) TAF, alone or in combination with hzVSF at an intermediate dose. The upper horizontal dotted lines represent the mean WHV cccDNA level of Groups 3–6 or Groups 7–8 at baseline, while the lower horizontal dotted lines indicate the detection limit for this molecule (2.0 pg/μg cellular DNA).

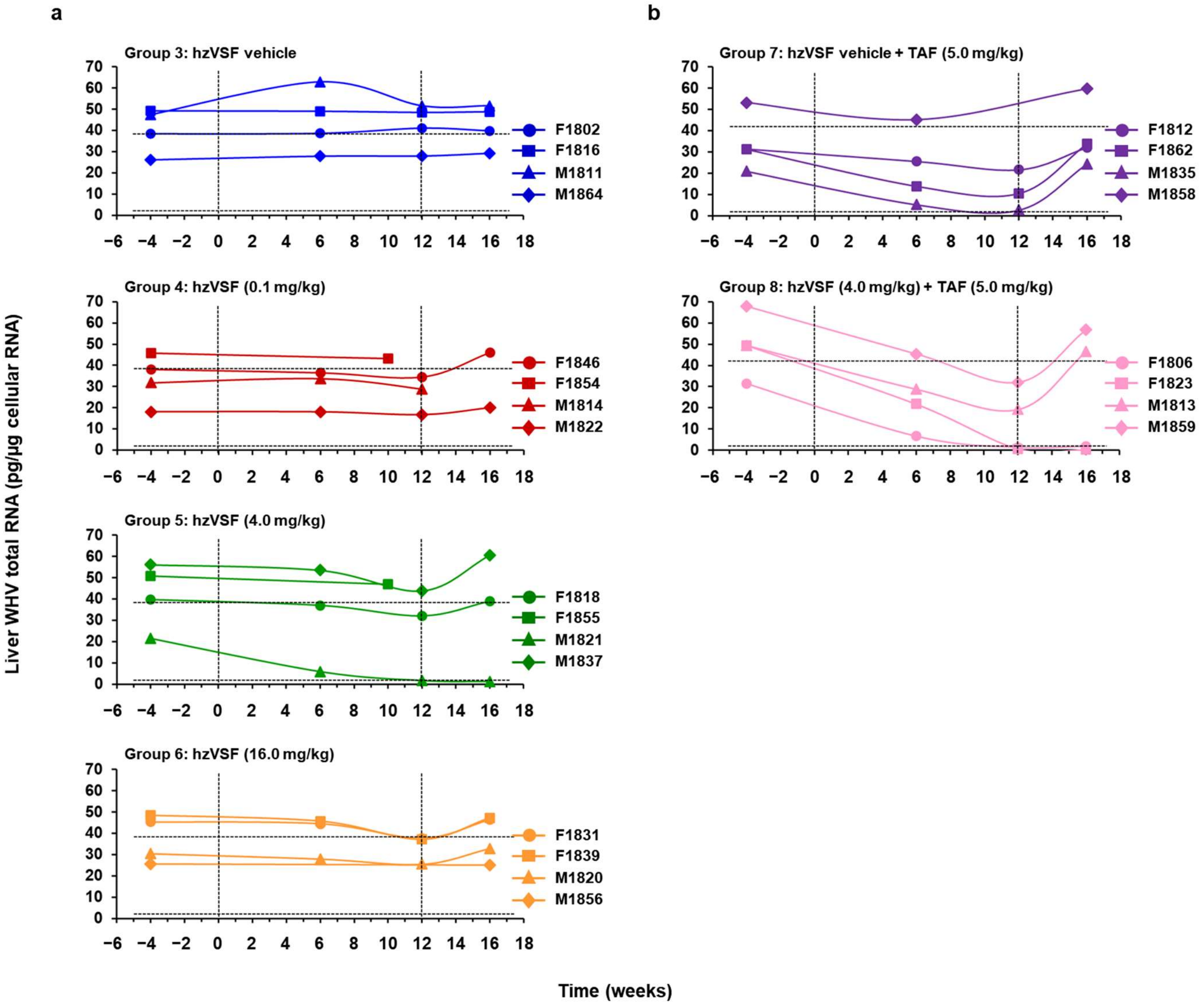

Figure S4. hzVSF minimally reduces WHV RNA in the liver, but suppression is pronounced in combination with TAF. Changes in intrahepatic WHV RNA level relative to the pretreatment baseline (week −4) of individual woodchucks treated with (**a**) placebo or hzVSF at a low, intermediate, and high dose and (**b**) TAF, alone or in combination with hzVSF at an intermediate dose. The upper horizontal dotted lines represent the mean WHV RNA level of Groups 3–6 or Groups 7–8 at baseline, while the lower horizontal dotted lines indicate the detection limit for this molecule (2.0 pg/µg cellular RNA).

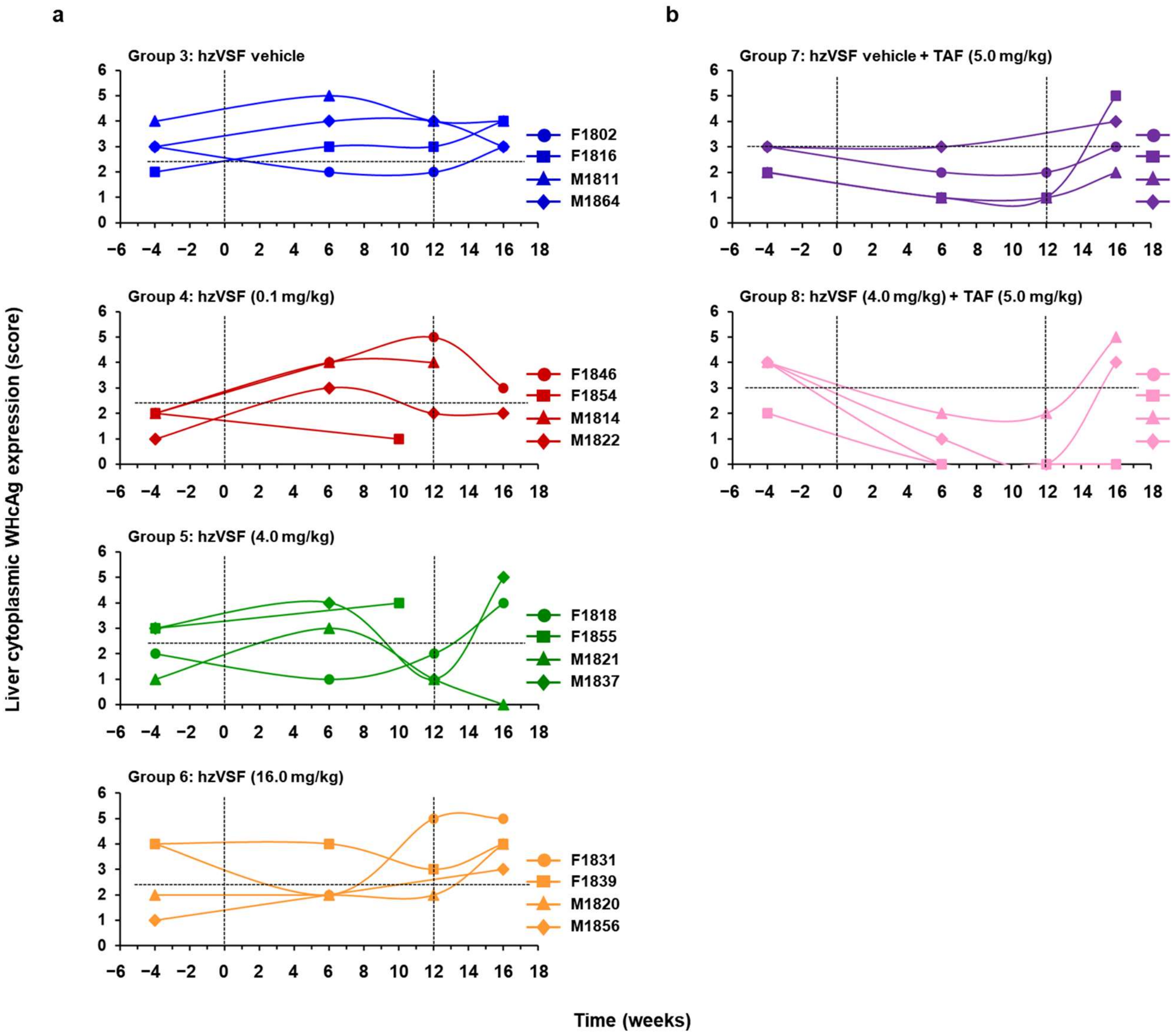

Figure S5. hzVSF does not reduce cytoplasmic WHcAg expression in the liver, but suppression is pronounced in combination with TAF. Changes in hepatic WHcAg expression relative to the pretreatment baseline (week −4) of individual woodchucks treated with (**a**) placebo or hzVSF at a low, intermediate, and high dose and (**b**) TAF, alone or in combination with hzVSF at an intermediate dose. Horizontal dotted lines present the mean WHcAg expression score of Groups 3–6 or Groups 7–8 at baseline. Score: 0 = 0%, 1 = 1–20%, 2 = 21–40%, 3 = 41–60%, 4 = 61–80%, and 5 = 81–100% of hepatocytes expressed WHcAg.

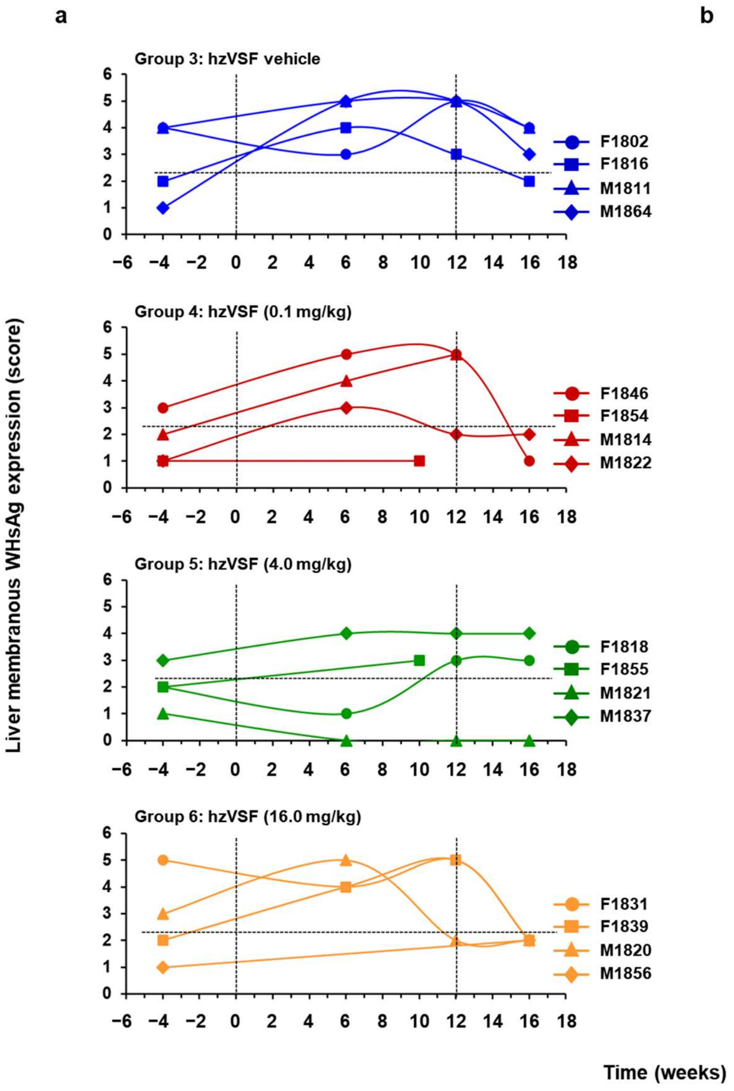

Figure S6. hzVSF does not reduce membranous WHsAg expression in the liver, but suppression is pronounced in combination with TAF. Changes in hepatic WHsAg expression relative to the pretreatment baseline (week −4) of individual woodchucks treated with (**a**) placebo or hzVSF at a low, intermediate, and high dose and (**b**) TAF, alone or in combination with hzVSF at an intermediate dose. Horizontal dotted lines present the mean WHsAg expression score of Groups 3–6 or Groups 7–8 at baseline. Score: 0 = 0%, 1 = 1–20%, 2 = 21–40%, 3 = 41–60%, 4 = 61–80%, and 5 = 81–100% of hepatocytes expressed WHsAg.

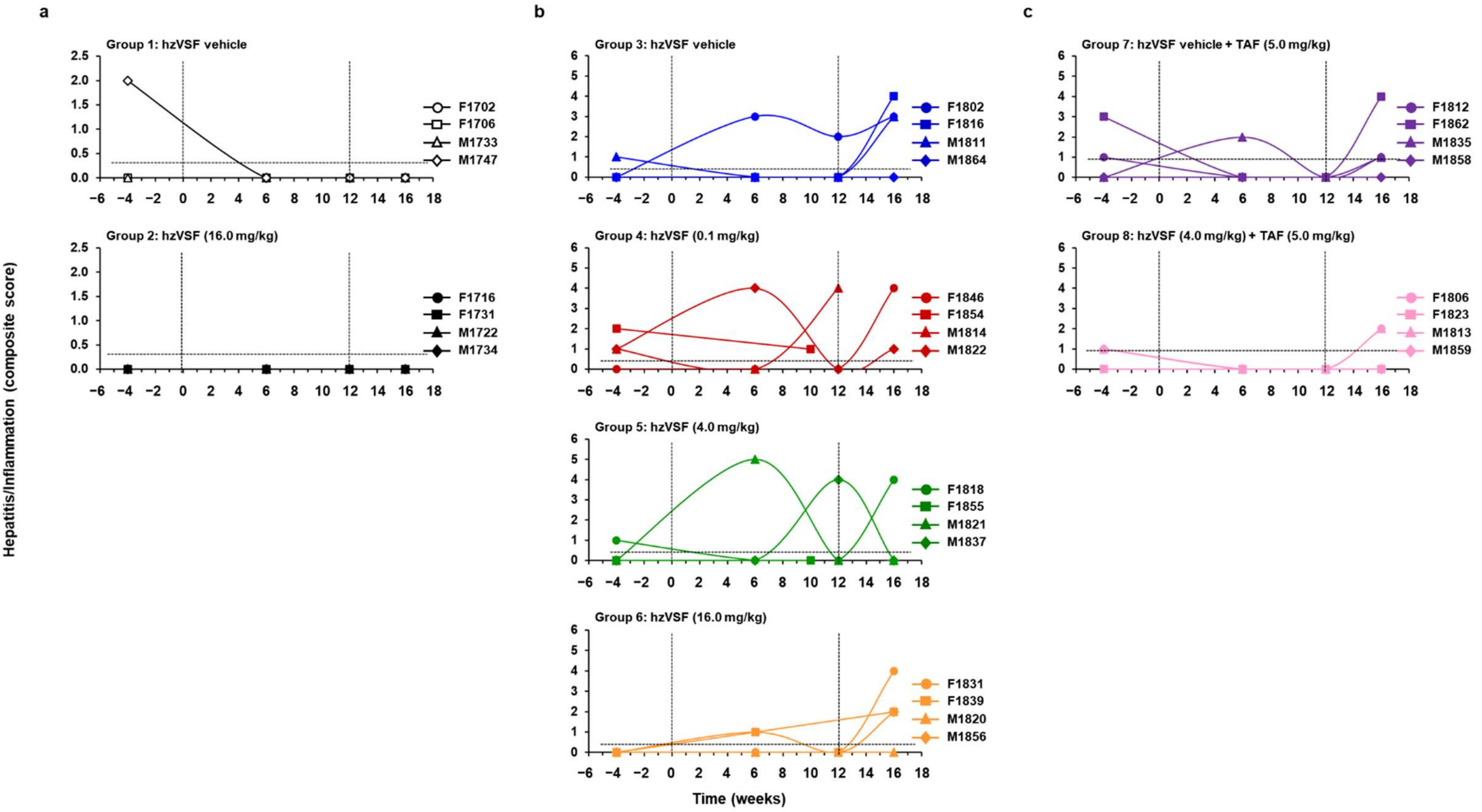

Figure S7. Treatment with hzVSF at the high dose or with TAF, alone and together with hzVSF at an intermediate dose, reduces inflammation in the liver. Changes in the composite score of portal and sinusoidal hepatitis relative to the pretreatment baseline (week −4) of individual WHV-uninfected woodchucks treated with (**a**) hzVSF vehicle or hzVSF at a high dose and of individual WHV-infected woodchucks treated with (**b**) hzVSF vehicle or hzVSF at a low, intermediate, and high dose and (**c**) TAF, alone or in combination with hzVSF at an intermediate dose. Horizontal dotted lines present the mean composite score of Groups 1–2, Groups 3–6, or Groups 7–8 at baseline. The composite score was derived from the sinusoidal hepatitis score plus the portal hepatitis score. Composite score: 0 = absent, >0–2 = mild, >2–4 = moderate, and >4 = marked to severe hepatitis/liver inflammation.

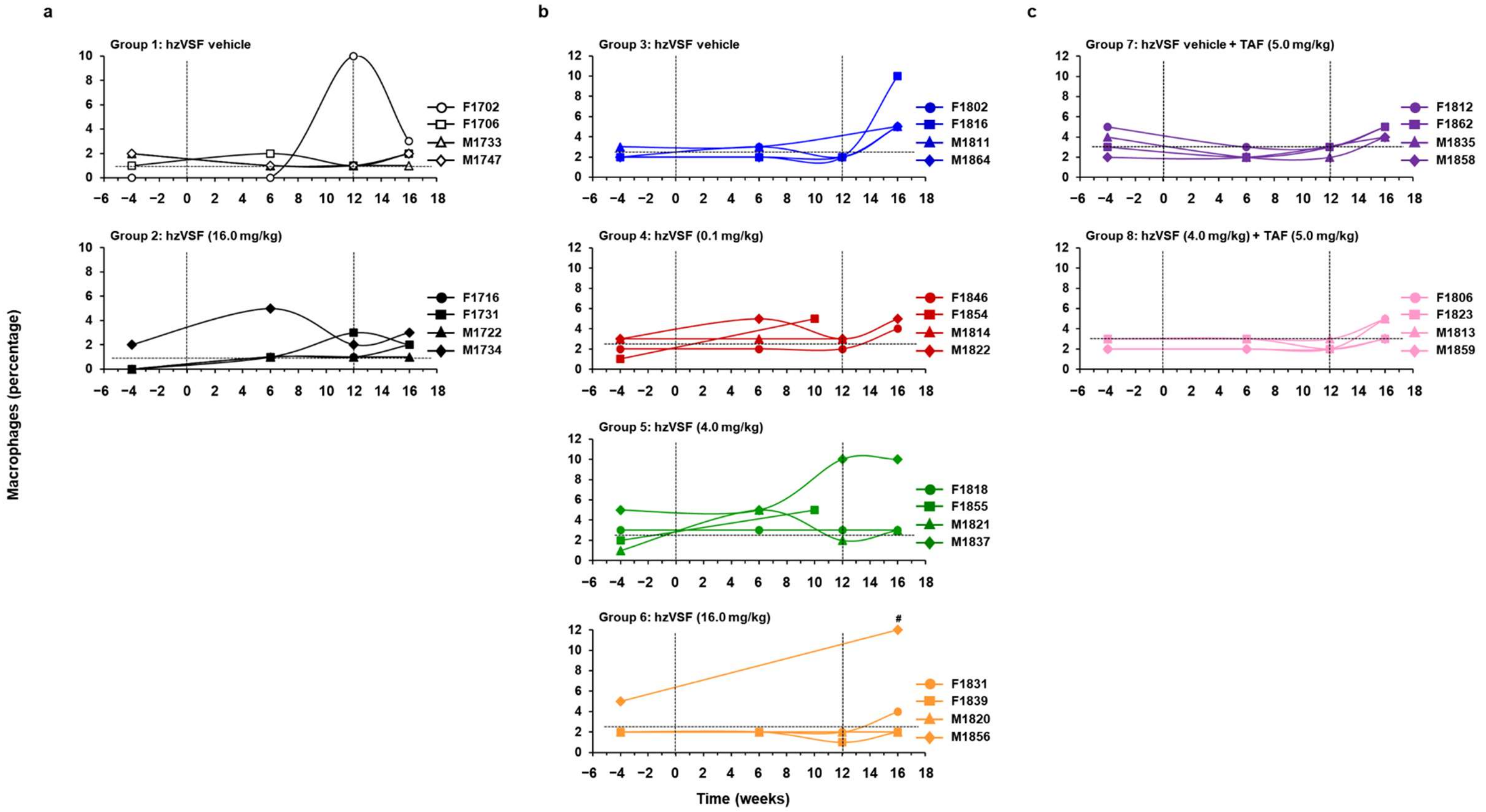

Figure S8. Treatment with hzVSF at the high dose or with TAF, alone and together with hzVSF at an intermediate dose, reduces the number of macrophages within liver. Changes in the intrahepatic macrophage percentage relative to the pretreatment baseline (week −4) of individual WHV-uninfected woodchucks treated with (**a**) hzVSF vehicle or hzVSF at a high dose and of individual WHV-infected woodchucks treated with (**b**) hzVSF vehicle or hzVSF at a low, intermediate, and high dose and (**c**) TAF, alone or in combination with hzVSF at an intermediate dose. Horizontal dotted lines present the mean macrophage percentage of Groups 1–2, Groups 3–6, or Groups 7–8 at baseline. # The macrophage percentage in liver of woodchuck M1856 (Group 6) was 20%.

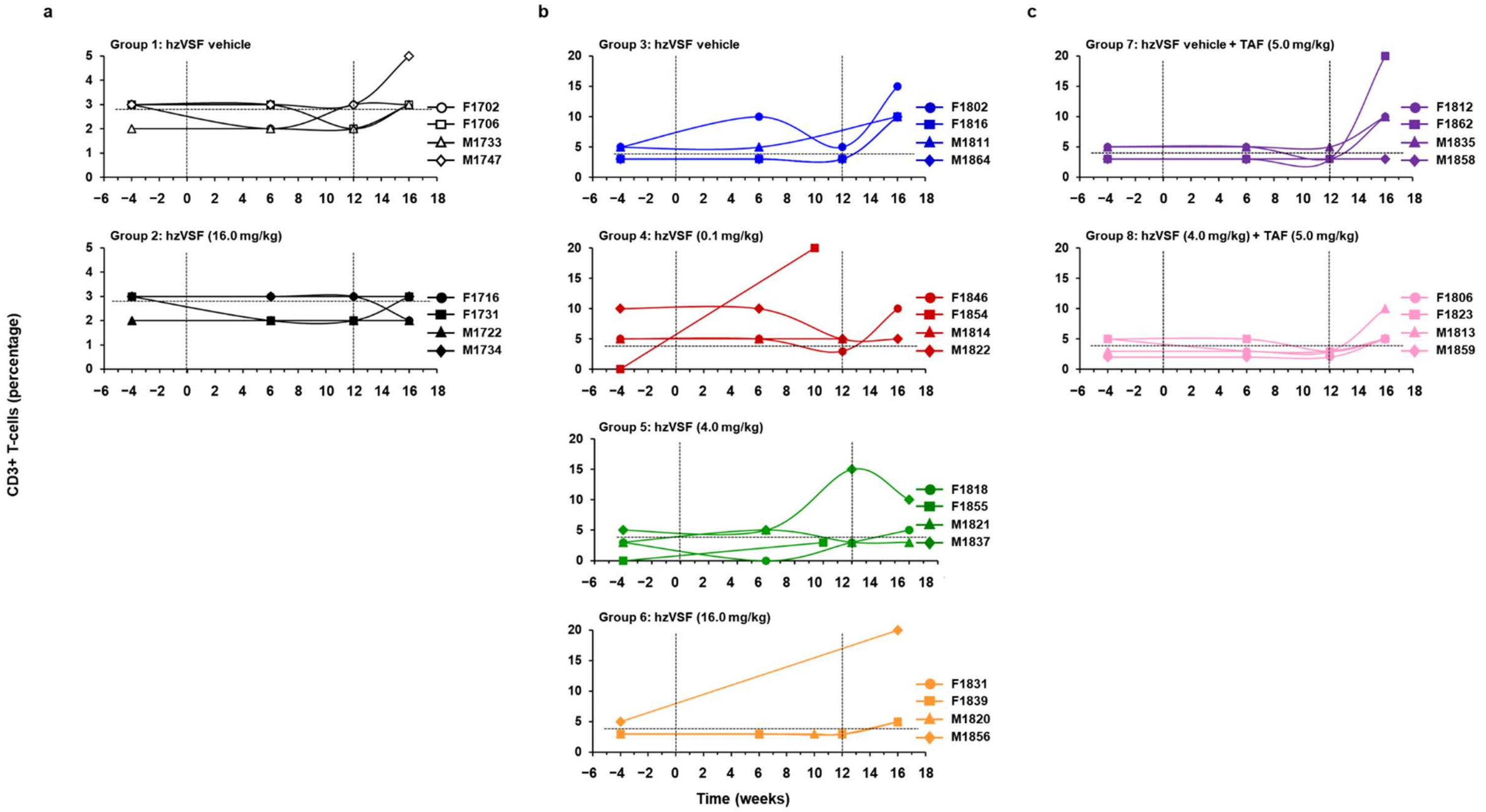

Figure S9. Treatment with hzVSF at the high dose or with TAF, alone and together with hzVSF at an intermediate dose, reduces the number of CD3+ T-cells within liver. Changes in the intrahepatic CD3+ T-cell percentage relative to the pretreatment baseline (week −4) of individual WHV-uninfected woodchucks treated with (**a**) hzVSF vehicle or hzVSF at a high dose and of individual WHV-infected woodchucks treated with (**b**) hzVSF vehicle or hzVSF at a low, intermediate, and high dose and (**c**) TAF, alone or in combination with hzVSF at an intermediate dose. Horizontal dotted lines present the mean CD3+ T-cell percentage of Groups 1–2, Groups 3–6, or Groups 7–8 at baseline.

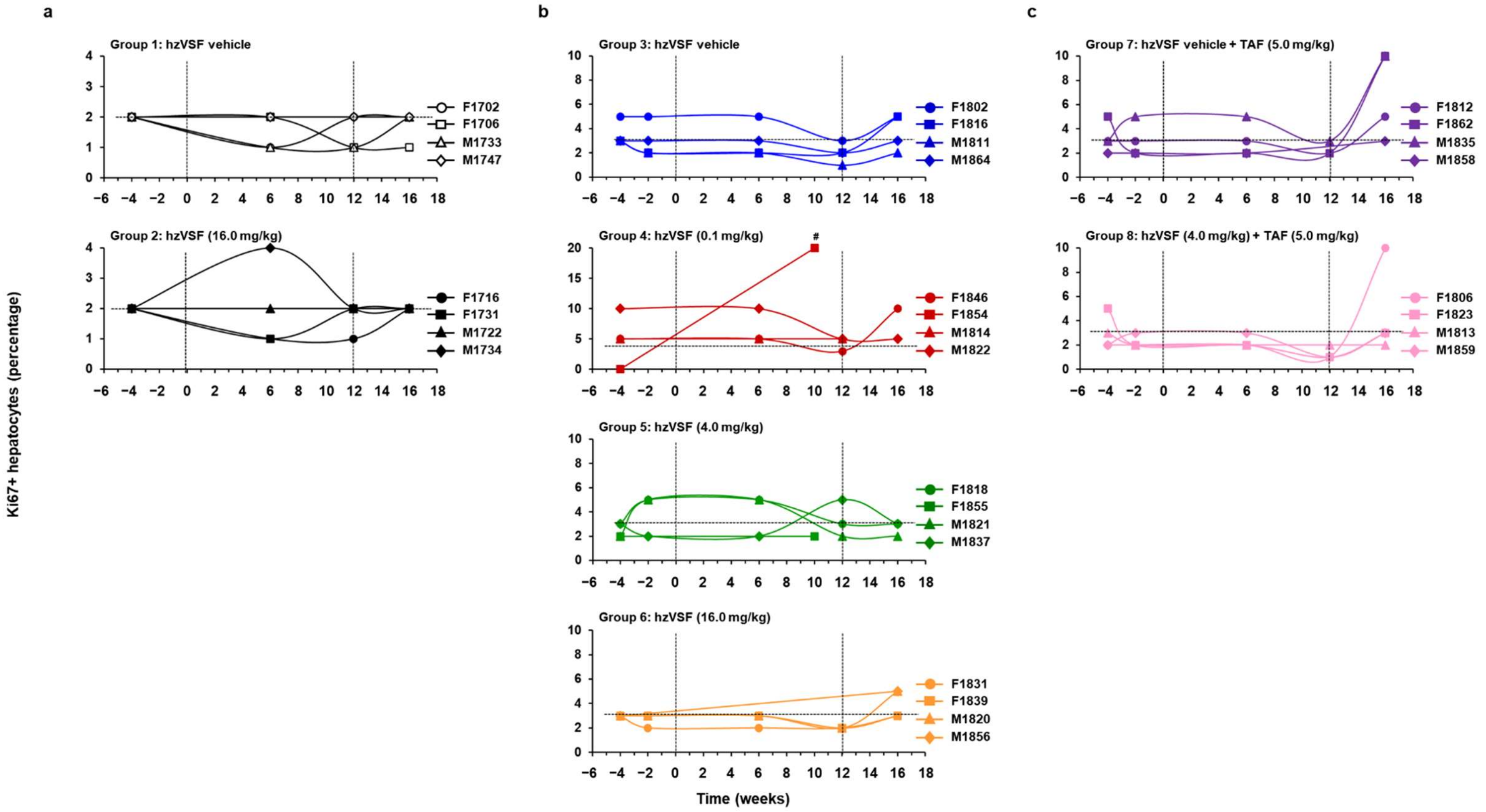

Figure S10. Treatment with hzVSF at the high dose or with TAF, alone and together with hzVSF at an intermediate dose, lowers liver replenishment by new hepatocytes. Changes in the Ki67+ hepatocyte percentage relative to the pretreatment baseline (week −4) of individual WHV-uninfected woodchucks treated with (**a**) hzVSF vehicle or hzVSF at a high dose and of individual WHV-infected woodchucks treated with (**b**) hzVSF vehicle or hzVSF at a low, intermediate, and high dose and (**c**) TAF, alone or in combination with hzVSF at an intermediate dose. Horizontal dotted lines present the mean Ki67+ hepatocyte percentage of Groups 1−2, Groups 3−6, or Groups 7−8 at baseline. # The Ki67+ hepatocyte percentage in liver of woodchuck M1822 (Group 4) was 30%.

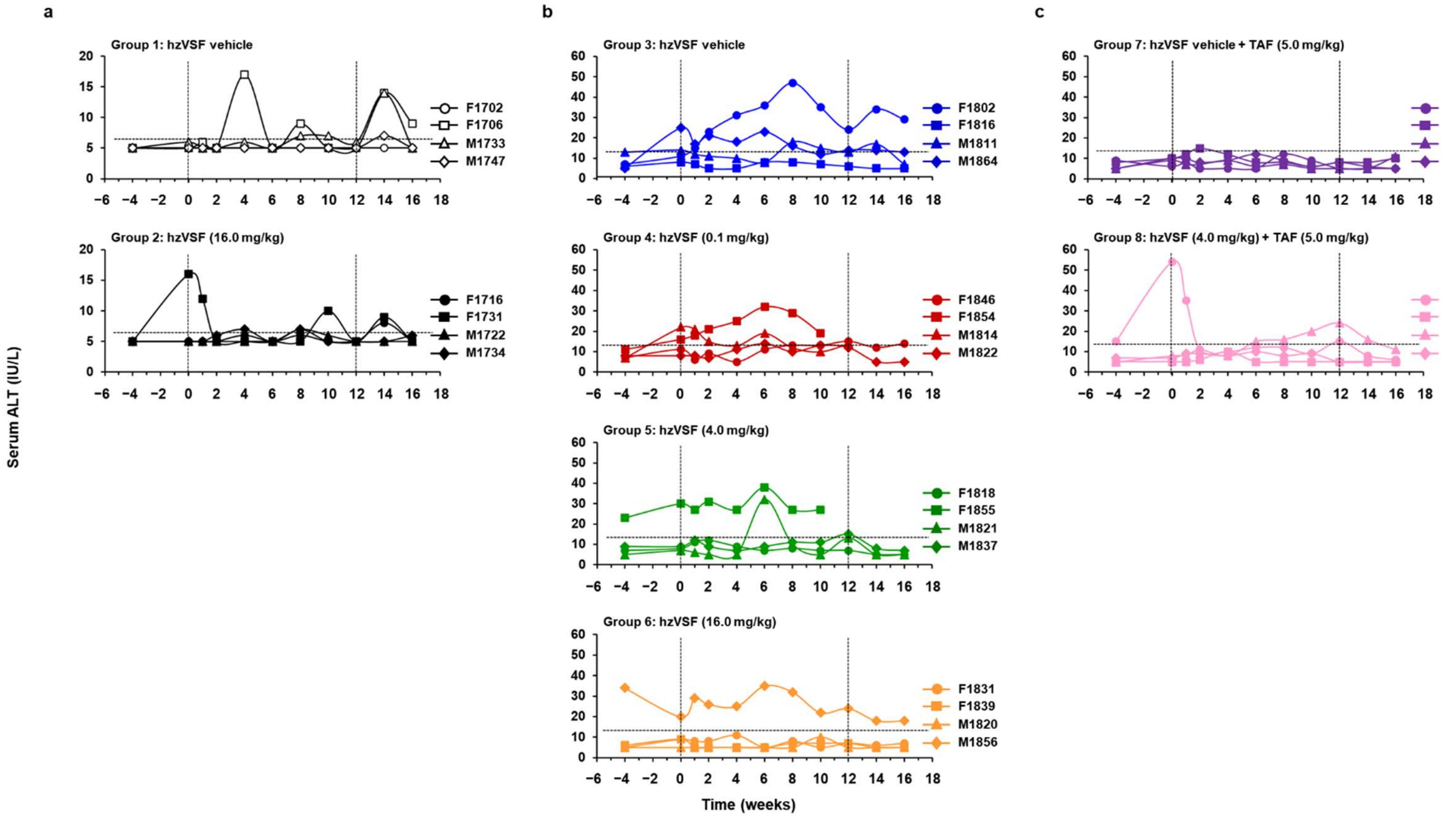

Figure S11. Treatment with hzVSF at the high dose or at an intermediate dose in combination with TAF does not induce elevations in serum ALT in most woodchucks. Changes in the serum ALT level relative to the pretreatment baseline (T0) of individual WHV-uninfected woodchucks treated with (**a**) hzVSF vehicle or hzVSF at a high dose and of individual WHV-infected woodchucks treated with (**b**) hzVSF vehicle or hzVSF at a low, intermediate, and high dose and (**c**) TAF, alone or in combination with hzVSF at an intermediate dose. Horizontal dotted lines present the mean ALT level of Groups 1–2, Groups 3–6, or Groups 7–8 at baseline. Abbreviation: IU, international unit.

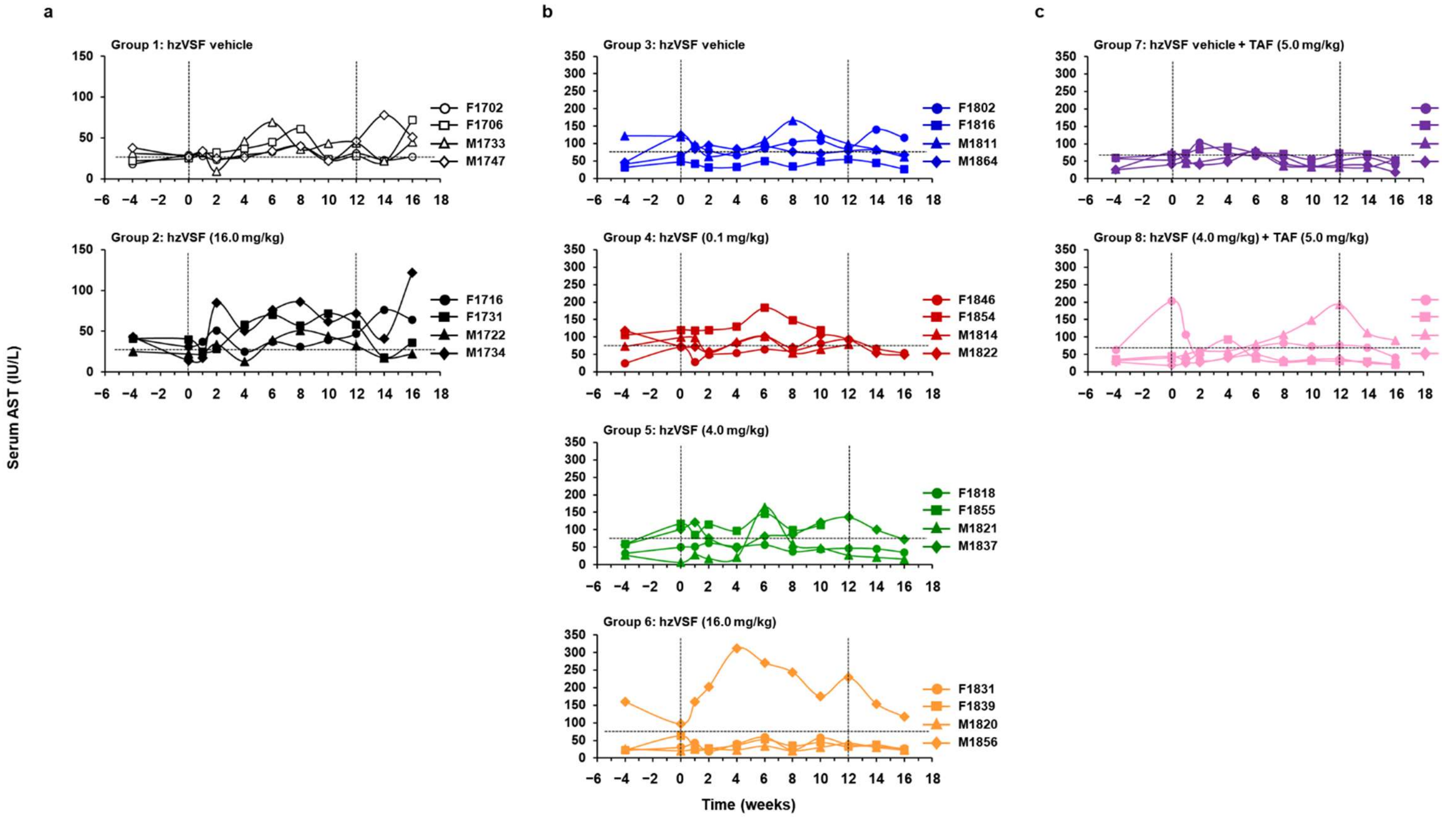

Figure S12. Treatment with hzVSF at the high dose or at an intermediate dose in combination with TAF does not induce elevations in serum AST in most woodchucks. Changes in the serum AST level relative to the pretreatment baseline (T0) of individual WHV-uninfected woodchucks treated with (**a**) hzVSF vehicle or hzVSF at a high dose and of individual WHV-infected woodchucks treated with (**b**) hzVSF vehicle or hzVSF at a low, intermediate, and high dose and (**c**) TAF, alone or in combination with hzVSF at an intermediate dose. Horizontal dotted lines present the mean AST level of Groups 1–2, Groups 3–6, or Groups 7–8 at baseline. Abbreviation: IU, international unit.

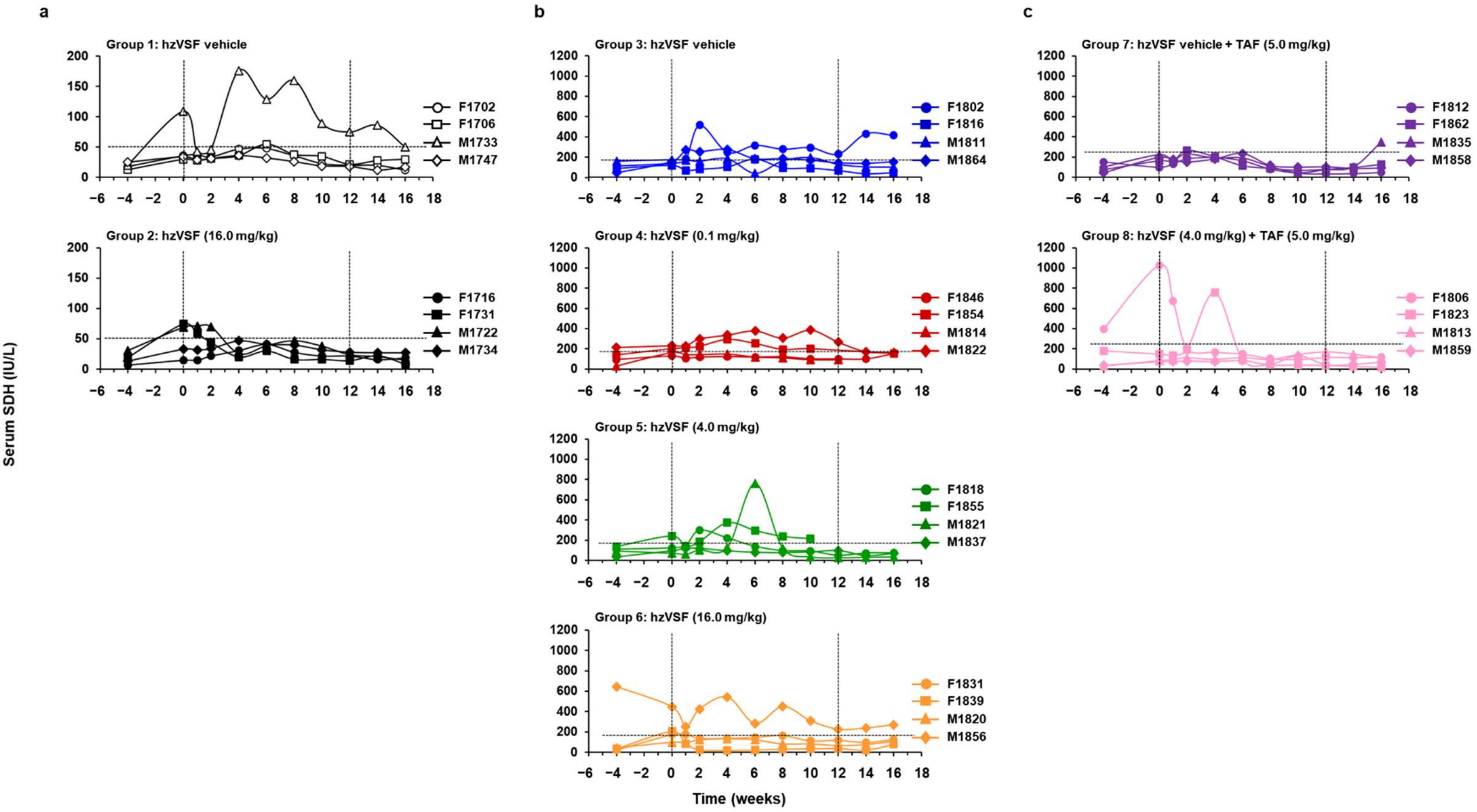

Figure S13. Treatment with hzVSF at the high dose or at an intermediate dose in combination with TAF does not induce elevations in serum SDH in most woodchucks. Changes in the serum SDH level relative to the pretreatment baseline (T0) of individual WHV-uninfected woodchucks treated with (**a**) hzVSF vehicle or hzVSF at a high dose and of individual WHV-infected woodchucks treated with (**b**) hzVSF vehicle or hzVSF at a low, intermediate, and high dose and (**c**) TAF, alone or in combination with hzVSF at an intermediate dose. Horizontal dotted lines present the mean SDH level of Groups 1−2, Groups 3−6, or Groups 7−8 at baseline. Abbreviation: IU, international unit.
